# Cecal Metabolomic Fingerprint of Unscathed Rats: Does It Reflect the Good Response to a Provocative Decompression?

**DOI:** 10.3389/fphys.2022.882944

**Published:** 2022-05-17

**Authors:** Anne-Virginie Desruelle, Sébastien de Maistre, Sandrine Gaillard, Simone Richard, Catherine Tardivel, Jean-Charles Martin, Jean-Eric Blatteau, Alain Boussuges, Sarah Rives, Jean-Jacques Risso, Nicolas Vallee

**Affiliations:** ^1^ Institut de Recherche Biomédicale des Armées, Equipe de Recherche Subaquatique Opérationnelle, Toulon Cedex, France; ^2^ Service de Médecine Hyperbare Expertise Plongée, Hôpital d'Instruction des Armées Sainte-Anne, Toulon Cedex, France; ^3^ Université de Toulon, Toulon Cedex, France; ^4^ C2VN, INRAE, INSERM, BIOMET, Aix Marseille University, Faculté de Médecine La Timone, Marseille, France

**Keywords:** decompression sickness, inflammation, dive, oxygen, oxydative stress, interleukine

## Abstract

On one side, decompression sickness (DCS) with neurological disorders lead to a reshuffle of the cecal metabolome of rats. On the other side, there is also a specific and different metabolomic signature in the cecum of a strain of DCS-resistant rats, that are not exposed to hyperbaric protocol. We decide to study a conventional strain of rats that resist to an accident-provoking hyperbaric exposure, and we hypothesize that the metabolomic signature put forward may correspond to a physiological response adapted to the stress induced by diving. The aim is to verify and characterize whether the cecal compounds of rats resistant to the provocative dive have a cecal metabolomic signature different from those who do not dive. 35 asymptomatic diver rats are selected to be compared to 21 rats non-exposed to the hyperbaric protocol. Because our aim is essentially to study the differences in the cecal metabolome associated with the hyperbaric exposure, about half of the rats are fed soy and the other half of maize in order to better rule out the effect of the diet itself. Lower levels of IL-1β and glutathione peroxidase (GPX) activity are registered in blood of diving rats. No blood cell mobilization is noted. Conventional and ChemRICH approaches help the metabolomic interpretation of the 185 chemical compounds analyzed in the cecal content. Statistical analysis show a panel of 102 compounds diet related. 19 are in common with the hyperbaric protocol effect. Expression of 25 compounds has changed in the cecal metabolome of rats resistant to the provocative dive suggesting an alteration of biliary acids metabolism, most likely through actions on gut microbiota. There seem to be also weak changes in allocations dedicated to various energy pathways, including hormonal reshuffle. Some of the metabolites may also have a role in regulating inflammation, while some may be consumed for the benefit of oxidative stress management.

## Introduction

With the development of metabolomic approaches, it is easier to have a global vision of biological events, without neglecting the interactions between the hosts. The number of the total microorganisms colonizing the human gastrointestinal tract is estimating at over 100 trillion (Turnbaugh et al., 2006). Self-regulation of the different microbial communities is possible (Cao et al., 2019) but interaction with the host is also true. Actually, the gut microbial community can benefit the host in many aspects (Zhang et al., 2020). Not only can it harvest energy, produce vitamins and modulate the metabolism of bile acids but also it can protect the host from pathogens, modulate host’s immune system and maintain the integrity of the intestinal barrier (Thursby and Juge, 2017; Enright et al., 2018; Zhang et al., 2018). Diet, delivery mode, ethnic origin, age and the use of antibiotics are some of the common factors which can affect the composition of the gut microbiota (Ianiro et al., 2016; Rutayisire et al., 2016; Deschasaux et al., 2018; Zmora et al., 2019; Albouery et al., 2020) and the dysbiosis of it may induce diseases of the immune system, the endocrine system, the cardiovascular system and the nervous system (Illiano et al., 2020; Wang et al., 2022). All of these systems are discussed in the various studies dealing with decompression sickness (DCS).

When working under pressure or in scuba diving, the inhaled gases dissolve in the organism tissues as pressure increases. When the desaturation is too fast, these gases may then give rise to an excessive quantities of bubbles in the organism (Bert, 1978). It is admitted that the number of venous bubbles is a positively correlated to the risk of DCS (Sawatzky and Nishi, 1991; Brubakk and Neumann, 2003). However, for a same dive profile, the intra- and inter-individual variability is high in terms of bubble quantity and occurrence of DCS. The historically identified risk factors are age (Ardestani et al., 2015) and overweight (Pollock, 2007). Hereditary traits, such as gender (Hagberg and Ornhagen, 2003; Buzzacott et al., 2014; Lautridou et al., 2017), can also influence the ability to escape DCS. Recently, the heritable component of DCS was able to be highlighted through the selection and then the breeding of rats resistant to DCS (Lautridou et al., 2017; Lautridou et al., 2020). Compared to the founding population, a reshuffle in fecal composition has been characterized in these animals and it was suggested a rearrangement of the microbial community in relation to the change of antibiotic-like compounds but also in sugar, vitamins and bile acids availability. The variation in the amounts of certain phytohormones and corticosteroids (de Maistre et al., 2020; Vallee et al., 2021) in the cecum also begs the question of homeostatic regulation more broadly. Actually, another study using metabolomics (de Maistre et al., 2020), and serving as a basis for this work, also raises this problem, knowing that thermoregulation problems are also observed in mice suffering from DCS (Desruelle et al., 2019). In these animal studies, antibiotic strategies are sometimes more or less satisfactory in warding off DCS (de Maistre et al., 2016; de Maistre et al., 2018; Desruelle et al., 2019). Several preconditioning methods were conducted with specific diet to fight against DCS or bubble formation (de Maistre et al., 2016; Germonpré and Balestra, 2017; Arieli, 2018; Bosco et al., 2018; de Maistre et al., 2018; de Maistre et al., 2020). Studies directly interested in the intestinal metabolome are rare or patchy (de Maistre et al., 2016a; de Maistre et al., 2016b; de Maistre et al., 2018; de Maistre et al., 2020; Vallee et al., 2021). However, all of these studies are interested in oxidative stress and inflammatory reactions directly or indirectly, because these two phenomena are listed in DCS but also during non-pathological dives. Because on the one hand hyperoxic conditions are common in diving and on the other hand ischemia is suspected in DCS, a wide range of strategies was tested (Yang et al., 2015; Vallée et al., 2016; Cosnard et al., 2017; Germonpré and Balestra, 2017; Lambrechts et al., 2018).

What interests us is that the fecal metabolome can also endure these special conditions of dive in the absence of DCS. Actually, we wonder how gastrointestinal tract of rats reacts when they escape a provocative-diving protocol. We ask this question because it is documented that oxidative stress is a crucial determinant of some bacterial community structure (Ashley et al., 2020) and also because a reshuffle in the fecal metabolome can be exploited by pathogens in competition with the resident microbiota (Rohmer et al., 2011). Since, in addition to the production of antimicrobial compounds by resident species, the intestinal microbial community can modulate bone marrow and spleen macrophage cytokine production to promote defense against intracellular microorganisms (Liévin-Le Moal and Servin, 2006; Endt et al., 2010), it is in our interest to improve understanding possible changes in the metabolome of rats that do not report symptoms of DCS.

The aim of this study is to determine whether there are differences between the fecal metabolome of rats that have withstood a risky dive, and that do not show the DCS clinical signs, and those who are not exposed to this hyperbaric protocol. The diving simulation protocol used in this study was designed to induce DCS (Pontier et al., 2008; Pontier et al., 2009; Pontier et al., 2011; Blatteau et al., 2013) and the animals studied here are those having resisted this aggressive decompression (i.e. about half are asymptomatic: NoDCS) compared to another group of rats (Ctrl) not subjected to this dive. Factually, it is difficult to compare the three groups of rats together since the comparison of the Ctrl and NoDCS groups informs about the dive (compression decompression and hyperoxia, … ) effects while the comparison of the Ctrl and DCS groups informs about the disease in addition to the dive effect. The comparison of the NoDCS and DCS groups is the subject of another document (de Maistre et al., 2020). The comparison of all these groups is discussed at the end of the document.

Moreover, insofar as the metabolome fingerprint is obviously a function of the initial diet, we use a diet based on maize (cereal) and another based on soy (legume) in order to take advantage of a contrast effect highlighting the effect of the accident-provoking hyperbaric exposure.

## Methods

### Animals and Ethical Statement

All procedures involving experimental animals follow the 3Rs and complies with European Union rules (Directive 2010/63/EU) and French law (Decree 2013/118). The Ethics Committee of the Institut de Recherche Biomédicale des Armées approved this study in 2016. Sprague-Dawley male rats (Harlan laboratory, France) are housed in an accredited animal care facility, at 22 ± 1°C. They are housed in cages both during rest and during the experiments and maintained on a regular day (6:00 a.m.–6:00 p.m.)/night (12 h) cycle. Before the beginning of the study, food (kibble from Harlan Laboratories, 18% protein) and water are provided *ad libitum*.

For monitoring the welfare of the animals, i.e., the stress and pain felt by each animal, a dedicated observer fills out a scorecard (Supplementary Data) inspired from the Swiss veterinary guide (Cosnard et al., 2017). The items refer to licking, vocalization, the presence of tears, labored breathing, aggression or withdrawn behavior, motor or locomotor disorders with paresis for example. 0 corresponds to no stress and 3 is the maximum. A degree of stress of 3 in one case or a total score of 12 represents a criterion for stopping the procedure. In this study, no score reaches 12, and it is not necessary to resort to anticipated euthanasia.

At the end of the experimentation, the animals are anaesthetized by induction with isoflurane (Bellamont, firstly at 5% then 2%), in order to minimize stress while saving time, then by intraperitoneal injection (1 ml syringe, Omnican, B. Braun, Melsungen, Germany) of a mixture of ketamine (Imalgene 1000, 100 mg/kg, AstraZeneca, London, United Kingdom), acepromazine (Calmivet, 1.65 mg/kg, Vétoquinol S.A., Lure, France) and xylazine (Rompun 2%, 16 mg/kg, Bayer HealthCare, KVP, Kiel, Germany).

### Batches and Food

Because our aim is to study the differences in the cecal metabolome associated with the hyperbaric exposure while distinguishing the effects of the diet itself, to better rule them out, 56 rats (300–325 g; 9–10 weeks old) are fed either legume (SOY) or cereal (MAIZE), at a rate of 30 g per day, 30 days before the hyperbaric exposure. 29 animals receive maize (MAIZE) and 27 are fed soy (SOY). The animals are fed by our technician and the rats are identified by a code unknown to the staff in charge of the physical examination.

**FIGURE 1:**
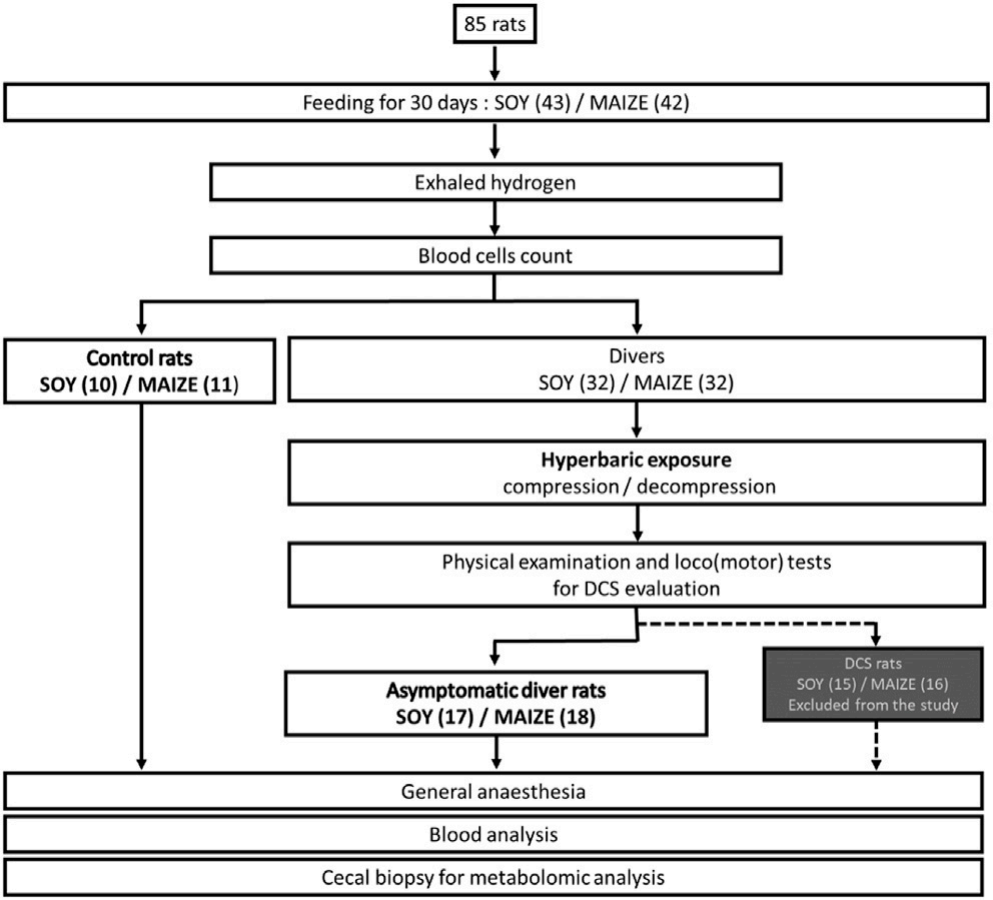
Flow chart process.

This article is part of a larger study on DCS (Figure 1) where the protocol used is specifically designed to induce accidents in some of the exposed rats and the animals studied here are those having resisted this provocative decompression (i.e., around half of asymptomatic NoDCS) and they are compared to another group of rats (Ctrl) not subjected to this dive. In the same way that it is not technically possible to follow the microbiota of a same rat before and after diving without greatly increasing the risk of diving accident, it is statically not possible to compare the three groups of rats together since the comparison of the Ctrl and NoDCS groups informs about the pressure effect while the comparison of the Ctrl and DCS groups informs about the disease in addition to the pressure effect. As for the comparison of the NoDCS and DCS groups, it is the subject of another paper.

Because our previous studies show that the incidence of DCS increase with the body mass of the animals (Blatteau et al., 2015), rats are sorted in order to keep the lightest in the group of divers and thus to minimalize decompression accident. Other rats are assigned to the control group. The establishment of this lightweight group aims to enable a biological assessment over a larger number of animals while limiting their number. For a same diet, this separation into two subgroups, between divers’ rats and controls, is necessary because the collection of the cecal content requires an anesthesia and a surgical operation incompatible with a hyperbaric exposure and a safe decompression stage. The experimental design is detailed in Figure 1. A selection effect of the protocol naturally takes place between the divers (DCS and NoDCS) and Ctrl groups whereas there is no difference between DCS and NoDCS rats (de Maistre et al., 2020). The day of the dive protocol, the weight (mean ± Standard Deviation) in the Ctrl group (403.6 ± 4.4 g) is significantly higher (n_Ctrl/Diver_ = 21/35, F = 23.48, *p* < 0.0001) than that in the Diver group (376.7 ± 3.4 g). In each of these two groups, weights are not statistically different between SOY and MAIZE rats (n_SOY/MAIZE_ = 27/29, W_SOY_ = 389.5 ± 3.8 g, W_MAIZE_ = 390.9 ± 3.9 g, F = 0.064, *p* = 0.801).

The clinic is established by another staff and the coding is only revealed afterwards for processing the results.

### Exhaled Hydrogen

Gut fermentation is evaluated by measuring exhaled hydrogen 1 hour before pressurization. This protocol is the same as in our previous study (de Maistre et al., 2020). Briefly, because of the diffusion throughout the body *via* the bloodstream, the amount of alveolar H_2_ is proportional to H_2_ resulting from the bacterial fermentation in the gut. The measurement of H_2_ presupposes that the breathing frequency of Sprague-Dawley rats is constant over time and that it approaches the value of 225 ml min^−1^ under stress (de Maistre et al., 2020), considering the literature (Gungor et al., 2016). For measurement, animal is placed in a clean cylinder (internal volume of 883 ml) allowing air circulation at constant flow rate of 225 ml ml.min^−1^ (aerator: Rena Air 200, France) and the collection of gases at the other end. 5 min time is necessary for homogenization of the gases inside the cylinder. Successive measurements of H_2_ (Gastrolyser, Respur International, France) in the air coming out of the cylinder are then performed.

### Hyperbaric Exposure

To remain comparable, we repeat the protocol dedicated of our previous experiences (Cosnard et al., 2017; de Maistre et al., 2018; Lambrechts et al., 2018; de Maistre et al., 2020). Batches of 8 freely-moving rats (4 per cage and 4 per group) are subjected to the hyperbaric protocol, which generates decompressions sickness, in a 200 L caisson with three observation portholes. The protocol has two compression speeds. The animals are subjected to an air compression procedure at a speed of 10 kPa min^−1^ up to an absolute pressure of 200 kPa (corresponding to a depth of 10 m of seawater); and then a speed of 100 kPa min^−1^ up to a pressure of 1000 kPa (corresponding to a depth of 90 msw) where they remain for 45 min. The rats are then decompressed at a speed of 100 kPa min^−1^ up to 200 kPa, and then a speed of 10 kPa min^−1^ until return to normal pressure, adhering to 5-min stages at 200 kPa (10 msw) and 160 kPa (6 msw) with a final stage of 10 min at 130 kPa (3 msw). The decompression speed is automatically controlled by a computer connected to an Analogue/Digital converter (NIUSB-6211, National Instrument, United States), itself connected to a solenoid valve (Belimo LR24A-SR, Switzerland) and a pressure transmitter (Pressure Transmitter 8314, Bürkert Fluid Control Systems, Germany). The program used to control the compression and decompression speeds is devised by a laboratory engineer at DASYLab (DASYLab National Instruments, United States). The compressed air is supplied by a diving compressor (Mini-Verticus III, Bauer Comp Holding, Germany) coupled to a 100-liter unit at 30 MPa, and connected to a pressure relief valve (LTHS 400 0086, ALPHAGAZ, Rousset, France). The oxygen analysis is performed using a micro-fuel electrochemical cell (G18007, Teledyne Electronic Technologies, Analytical Instruments, United States). The CO_2_ produced by the animals is captured with soda lime (<300 ppm, GE Healthcare, Helsinki, Finland). The gases are mixed by a fan, and the temperature inside the caisson is measured with a heat probe (Pt 100, Eurotherm, France).

### Physical Examination and Behavioral Tests

In order to ensure the good health of the animals and to be certain of the selection of No-DCS animals, a physical examination, identical to the previous one (Cosnard et al., 2017; de Maistre et al., 2020), is conducted by the main experimenter over a 30-min observation period following the end of the dive. A collection of clinical signs is conducted where the motor disorders, convulsions, respiratory difficulties, and death are referenced with a time index. These observations are assessed by: the Motor Performance Score (MPS), from 10 to 0, including specific tests for (loco)motor disorders (von Euler et al., 1996); the *beam-walk test* from 1 to 7 where the rat has to move on an ever-narrower board, from 7.7 to 1.7 cm, above the void; the *rollover test* (score from 0 to 2) consists of a simulated fall situation causing a reflex rollover in the rat so that it fell on its paws; the *toe-spreading reflex test* (Pockett and Gavin, 1985) assesses a functional impairment of the sciatic nerve (SFI Index), by a visual observation of the toe-spreading where 0 is a complete inability to spread the toes, 1 a weak spread, and 2 a normal state; the diagnosis of motor impairment of the hind paws (MIHP) come in addition to the SFI and scores at 0 an inert paw, 1 a paw that no longer moves but is still capable of muscle contraction, 2 a paw spontaneously to the rear, 3 a paw which is stretched and does not go back into place spontaneously, 4 a rat which limps, and 5 a normal motricity. Decompression accident is pronounced as far as the rat is presenting neurological signs in the form of paresis or paralysis of at least one limb, convulsions and/or reduced performance in SFI, MIHP locomotor tests, with a *beam walk test* score reduced by at least 2 points. These symptomatic rats are not considered in this study (DCS occurrence: Maize/Soy *n* = 14/15) but in another one (de Maistre et al., 2020). The other rats are considered asymptomatic, and they are included in this study (Maize/Soy *n* = 18/17).

### Anesthesia and Sacrifice

Thirtyminutes after the physical examination, the animals are anesthetized by induction with isoflurane (Bellamont, firstly at 5% then 2% in 100% air flow at 2.0 L/min), then by intraperitoneal injection (1 ml syringe, Omnican, B. Braun, Melsungen, Germany) of a mixture of ketamine (Imalgene 1000, 100 mg/kg, AstraZeneca, London, UK), acepromazine (Calmivet, 1.65 mg/kg, Vétoquinol S.A., Lure, France) and xylazine (Rompun 2%, 16 mg/kg, Bayer HealthCare, KVP, Kiel, Germany). Anaesthesia level is determined by testing the lack of withdrawal reflexes in response to pinches of the distal hind limbs. At the end of the experiment, rats are sacrificed by an injection of sodium pentobarbital (200 mg/kg IP; Sanofi, Paris, France).

### Blood Analyses

We used our previously described protocol (de Maistre et al., 2016; Cosnard et al., 2017; de Maistre et al., 2018; Lambrechts et al., 2018; de Maistre et al., 2020). Briefly, the blood counts are performed from 15 µl blood taken from the tip of the tail and diluted in the same volume of 2 mM EDTA (Sigma, France). The analysis is performed using an automaton (Scil Vet abc, SCIL Animal Care Company, France) on samples taken 60 min before or 30 min after exposure to the hyperbaric protocol. The values for the second blood sample are corrected depending on the variation in the hematocrit.

### Cytokine Detection

We reproduce our analysis protocol (de Maistre et al., 2016; Cosnard et al., 2017; de Maistre et al., 2018; Lambrechts et al., 2018; de Maistre et al., 2020). Under anesthesia following the hyperbaric exposure, blood samples are collected by an intra-aortic puncture to determine the values of plasmatic cytokine levels. Blood is collected in sterile 4 ml tubes containing lithium heparin (BD Vacutainer, BD-Plymouth, UK) and, within 30 min, plasma is separated out by simple centrifugation at 1200 g and 4°C for 15 min. The supernatant is kept at −80°C until testing.

The pro-inflammatory cytokine Interleukine-1β (IL-1β) and oxidative stress markers Thiobarbituric Acid Reactive Susbtances (TBARS) and glutathione peroxidase (GPX) are assayed using a rat ELISA kit (ELISA Kit, Antibodies-Online GmbH, Germany) and QuantiChrom TBARS Assay Kit and EnzyChrom Glutathione Peroxidase Assay Kit (BioAssay Systems, CA, United States). Samples, standards, and quality controls are all run in duplicate. All standards and quality controls are made up as recommended by the supplier. It includes normalized data.

### Fecal Metabolome

This technique is similar to that previously used in our studies (de Maistre et al., 2020). Under anaesthesia, the cecum is separated from the digestive tract after ligation. Its content is kept at −80°C until metabolomic analysis.

100–150 mg of cecal content are homogenized in cooled methanol (3 μL/mg feces) at −20°C. Samples are vortexed for 1 min and incubated at −20°C for 30 min. Samples are then centrifuged for 15 min (11,000 x g, 4°C). The supernatant recovered from each sample is filtered through 10 KDa filter tubes by centrifuging for 45 min (11,000 x g, 4°C). The extracts obtained are then dried using a stream of nitrogen and then frozen at -80°C.

Liquid chromatography mass spectrometry (LCMS) metabolomic analyses are performed essentially as described earlier (Rosique et al., 2019). All the dried polar extracts are first reconstituted with 150 µl acetonitrile/water (50:50; v:v). The samples are separated using high performance liquid chromatography (UPLC) ultimate 3000 (Thermo Scientific), coupled to a high-resolution Q-Exactive Plus quadrupole-orbitrap hybrid mass spectrometer (HRMS), equipped with electrospray ionization source (H-ESI II). The chromatographic separation is performed on a binary solvent system using a HILIC column (Merk, SeQuant^®^ ZIC®-HILIC, 150 mm × 2.1 mm, 5 μm, 200 A) at 25°C with a flow rate of 0.25 ml min-1. The injection volume is 5 μl. The mobile phase consists of a combination of solvent A (100% water, 16 mM ammonium formate) and solvent B (100% acetonitrile 0.1% formic acid). The following gradient conditions are used: 0–2 min, isocratic 97% B; 2–10 min, linear from 97 to 70% B; 10–15 min, linear 70 to 10% B; 15–17 min, isocratic 10% B; 17–18 min linear from 10 to 97% B; from 18 to 22 min isocratic 97% B. The separated molecules are analyzed in both positive and negative ionization modes in the same run (switch mode). The signal/noise ratio used is 5. The repeatability of the analysis is checked by analyzing interspaced (1 out of every 5 samples) quality control samples (QC).

Data processing and molecule identification: All the raw data generated by the LCMS are converted to mzXML by ProteoWizard (Version 2.0), and then processed by MZmine 2.26. The identification of the metabolites is performed by using an in-house database referencing more than 800 metabolites with their chromatographic retention time acquired with a HILIC column, together with their exact mass and MSMS spectra obtained in positive and negative ionization modes, including their adducts and neutral losses. These leaves to Level 1 (MSMS, retention time, MS) or 2 identification (retention time, MS).

The MS metabolomics data from the 2 ionizations mode are merged into a single dataset after discarding duplicated ions, together with the other corresponding biological data and microbiota measurements, giving rise to 226 variables per rat.

### Statistical Analyses

Statistical analyses are performed essentially as described in our previous study (de Maistre et al., 2020). Most series of values fall between 0 and 1 and the distribution is positively skewed. Prior analysis, scale-contracting transformation is applied with log(X + 1). The difference is analyzed using 2-way ANOVA (type III SS) on clinical status and diet, comprising interactions, followed by post-hoc Tukey’s (HSD) and Benjamini–Hochberg’s (False Rate Discovery) tests. Principal component analysis (Pearson correlation coefficient), ascending Hierarchical Classification (AHC) (dissimilarity; Euclidean distance; Ward’s method) helped by k-means clustering are used to design the heat map and volcano plot, from normalized data of the 226 features of the 56 rats. A random forest classifier (Sampling: random with replacement; method: random input) is also used in order to extract the most contributing compounds. The software is XlStat Biomed from Addinsoft. Maximum accepted alpha level was 5%.

## Results

### Clinical Observation

The hyperbaric protocol indeed generates decompression sickness. Respectively, 53% (*n* = 17) and 56% (*n* = 18) of rats fed soy or maize are asymptomatic, implying the highest scores on behavioral and (loco)motor tests. The incidence of clinical signs of neurological DCS between these groups of rats is therefore not significantly different (*n* = 32/32, *p* = 0.806). Including 21 rats (n_SOY/CORN_ = 10/11) not exposed to the decompression protocol, 56 asymptomatic animals are selected for this study. 29 symptomatic animals are analysed in another study (n_SOY/CORN_ = 15/16).

### Blood Analysis Exhaled Hydrogen Analysis

**FIGURE 2:**
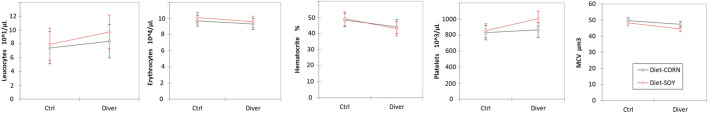
Blood cells count in Control and Diver rats, according to their diet (MAIZE or SOY).

Before the dive, the SOY rats have more platelets than MAIZE rats (mean ± SD; PLA_MAIZE_ = 423 ± 14 .10^3^/µl; PLA_SOY_ = 465 ± 14.10^3^/µl; F = 4.28, *p* = 0.042). Before the dive, the Ctrl rats have less platelets than Divers rats (mean ± SD; PLA_Ctrl_ = 421 ± 16 .10^3^/µl; PLA_Div_ = 467 ± 12.10^3^/µl; F = 5.29, *p* = 0.025). There is no interaction (F = 2.08, *p* = 0.156). Wilcoxon’s tests do not show any change in PLA counts after diving, whatever the group considered (Before/After PLA _Div SOY+MAIZE;_ Wilcoxon, *n* = 35, *p* = 0.359; Before/After PLA_Div SOY;_ Wilcoxon, *n* = 17, *p* = 0.579; Before/After PLA _Div MAIZE;_ Wilcoxon, *n* = 18, *p* = 0.468) (Figure 2).

The red corpuscles are smaller in the SOY rats (MCV_SOY_ = 46.4 ± 0.5 µm^3^; MCV_MAIZE_ = 48.6 ± 0.5 µm^3^; F = 8.52, *p* = 0.005). The red corpuscles are higher in the Ctrl rats (MCV_Ctrl_ = 49.0 ± 0.6 µm^3^; MCV_Div_ = 46.0 ± 0.4 µm^3^; F = 16.14, *p* = 0.0002). There is no interaction (F = 1.05, *p* = 0.310). No change occurrs after the protocol (Before/After MCV_Div SOY+MAIZE;_ Wilcoxon, *n* = 35, *p* = 0.188; Before/After MCV_Div SOY;_ Wilcoxon, *n* = 17, *p* = 0.161; Before/After MCV _Div MAIZE;_ Wilcoxon, n = 18, *p* = 0.716) (Figure 2).

No other blood cell count difference or mobilization is observed neither related to diet, nor to pressure exposure.

**FIGURE 3:**
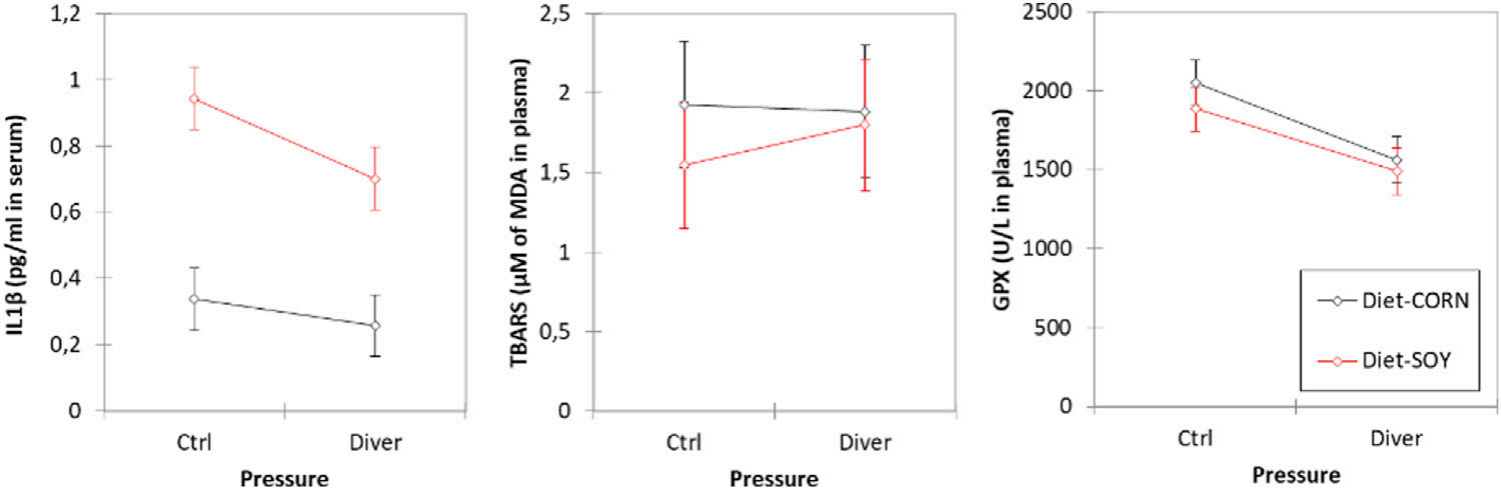
Inflammation and oxidative stress markers in plasma of Control and Diver rats, according to their diet (MAIZE or SOY): IL-1β, TBARS and GPX activity evaluation.

The average level of IL-1β is higher in SOY rats (IL-1β_SOY_ = 0.82 ± 0.03 pg/ml; IL-1β_MAIZE_ = 0.29 ± 0.03 pg/ml; F = 159.82, *p* < 0.0001). Levels of IL-1β are higher in Ctrl rats than those of Divers rats after the dive (IL-1β_CTRL_ = 0.64 ± 0.03 pg/ml; IL-1β_Diver_ = 0.48 ± 0.02 pg/ml; F = 15.03, *p* = 0.0003). There is no interaction (F = 3.78, *p* = 0.057) (Figure 3).

No significance is noted for TBARS, whatever is the group.

Levels of GPX are higher in Ctrl non-diving rats than those of Divers rats (i.e., after the dive) (GPX_CTRL_ = 1968 ± 50 U/L; GPX_Diver_ = 1525 ± 39 U/L; F = 47.11, *p* < 0.0001). Concerning the diet, no significance is noted for GPX (F = 3.36, *p* = 0.073) (Figure 3).

### Exhaled Hydrogen Analysis Before Dive

**FIGURE 4:**
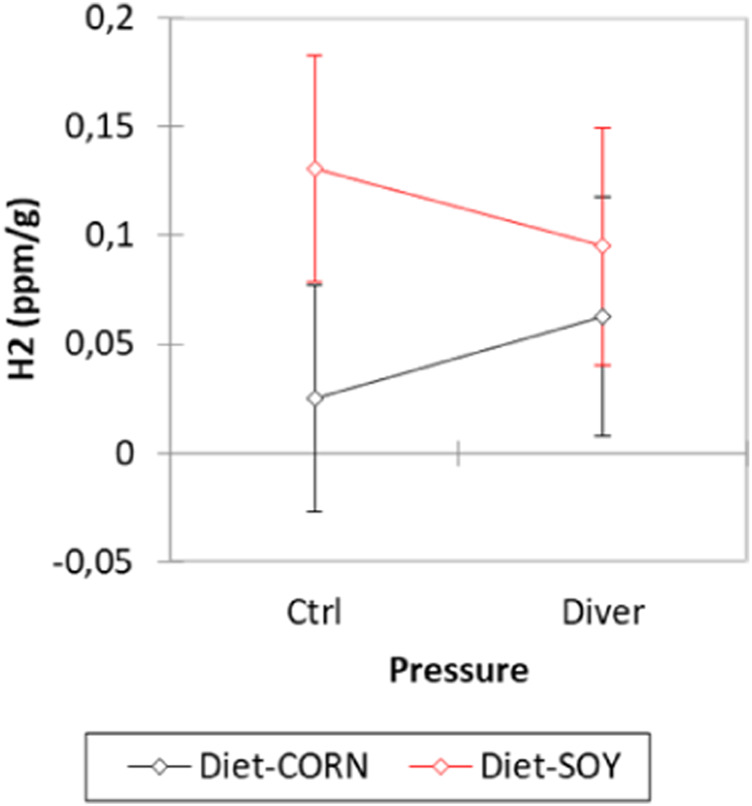
Exhaled hydrogen analysis before the dive from Control and Diver rats, according to their diet (MAIZE or SOY).

Before the dive, the level of exhaled hydrogen is higher in the SOY rats (H2_SOY_ = 0.113 ± 0.017 ppm/g; H2_MAIZE_ = 0.044 ± 0.016 ppm/g; F = 8.39, *p* = 0.0054). Concerning the pressure groups, no difference is noted (F = 0.003, *p* = 0.958) (Figure 4).

### Metabolomic Analysis of Feces

**FIGURE 5:**
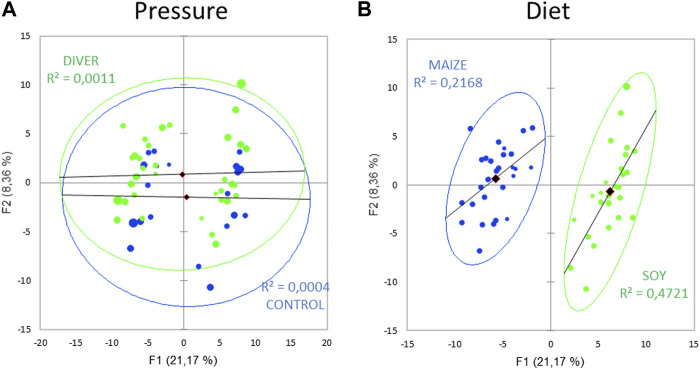
Principal component analysis (PCA) plot of fecal metabolome as function of **(A)** the exposure to pressure (Ctrl and Diver) or **(B)** the diet (maize or soy).

185 metabolites are analyzed in 56 rats (21 control and 35 asymptomatic rats). Principal component analysis (PCA; Pearson test) (Figure 5) of the fecal metabolome to reveal a diving effect shows two overlapped groups. A similar PCA highlights two distinct groups linked to diet, where the axes F1 and F2 explain 29% of the variability.

**FIGURE 6:**
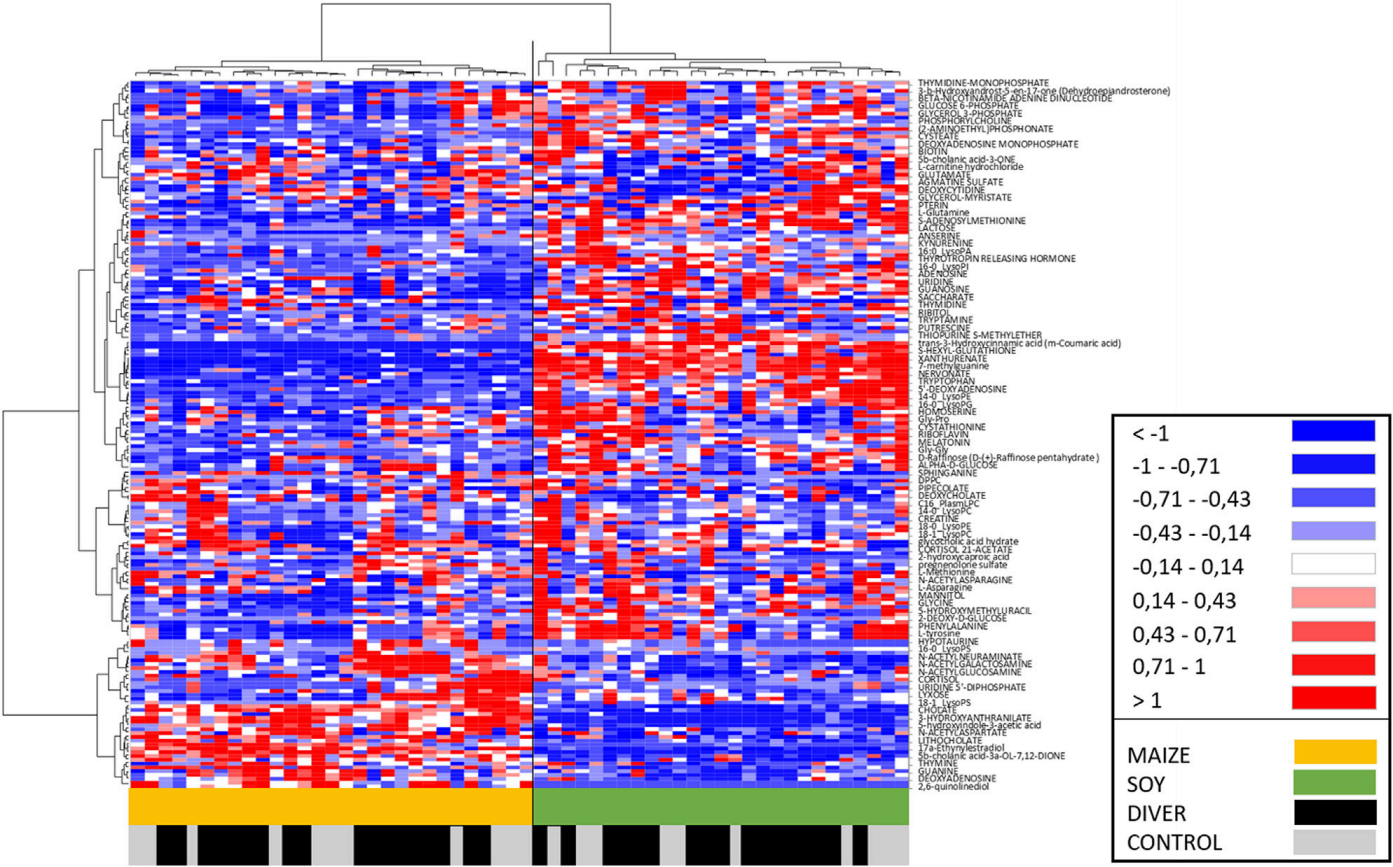
Heat Map, i.e., hierarchical clustering of fecal metabolomes from Ctrl (grey) or Diver (black) rats. Rats are on the ordinate where green notes rats fed with soy and orange those fed with maize. Fold-change for each metabolite (abscissa) is represented by a color. Intensity values are normalized, from red (Min: −1) to blue (Max: +1).

An Ascending Hierarchical Classification (AHC) are carried out as part of the Heat Map established according to the intensity of the 185 compounds analyzed (Figure 6). Following the evolution of the variances according to the number of classes, no inflection point clearly appears from the 2nd to the 6th class. The division into two classes explains 82% of the intragroup variability. According to the first division, a clear dichotomy is revealed with in the first class (I) all SOY rats (*n* = 27), while the second class (II) contains only MAIZE rats (*n* = 29). Divers and Ctrl are therefore equally distributed on both sides. The division into 3 classes explains 78% of the intragroup variability. Its first class Ia contains 25 SOY rats, and the second class (Ib) contains only 2 SOY rats (one DIVE and one CTRL). The class II remains unchanged with all MAIZE rats. The division into 4 or 5 classes involves subdivisions of the Ia class and it explains 75% or 72% of the intragroup variability, respectively. The division into 6 classes, explaining 70% of the intragroup variability, implies a sub-division of the MAIZE group (II). Whether the splits are in 2 or 6 classes, at no time is a dichotomy clearly made between Ctrl and Divers.

The AHC of the Heat Map allows to visualize on the abscissa a separation of the metabolites between the MAIZE rats on the left (orange) and the SOY rats on the right (green). In accordance with the previous AHC, no dichotomy is clearly made between Ctrl and Divers.

**FIGURE 7:**
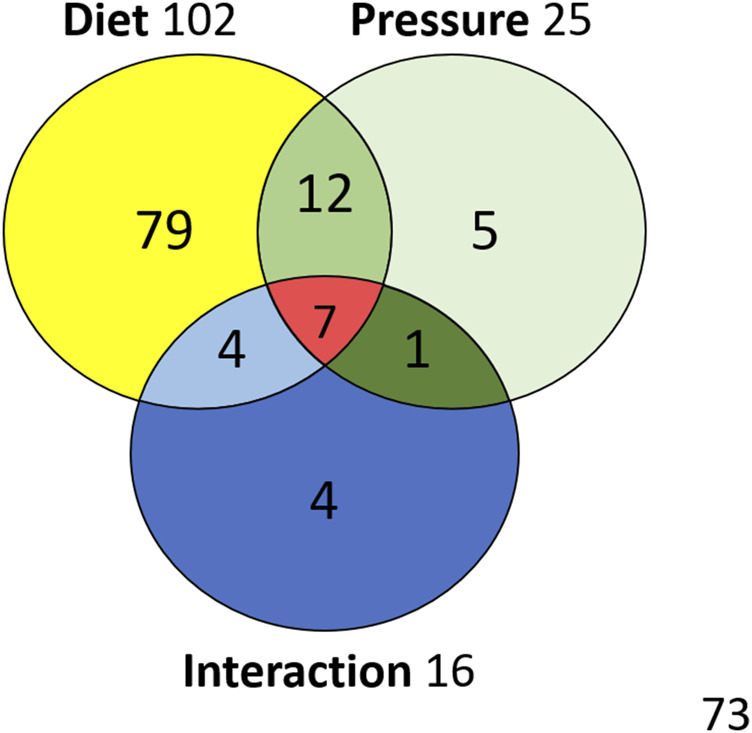
Venn diagram with metabolites influenced by diet or exposure to pressure, and their interactions.

The ANOVA [Type III SS with post-hoc Tukey (HSD) and Benjamini–Hochberg (FRD)] with two factors (Diet and Pressure) are performed for the 185 compounds in the 56 rats. The Venn diagram (Figure 7 + Supplementary Data 1 for the list of metabolites) allows to resume the effects linked to diet or pressure, as well as their interactions. Out of the 185 compounds analyzed, 102 are significantly influenced by the diet and 25 differ after a pressure exposure. 19 interactions are noted including 7 synergies. Five are specific to diving.

**FIGURE 8:**
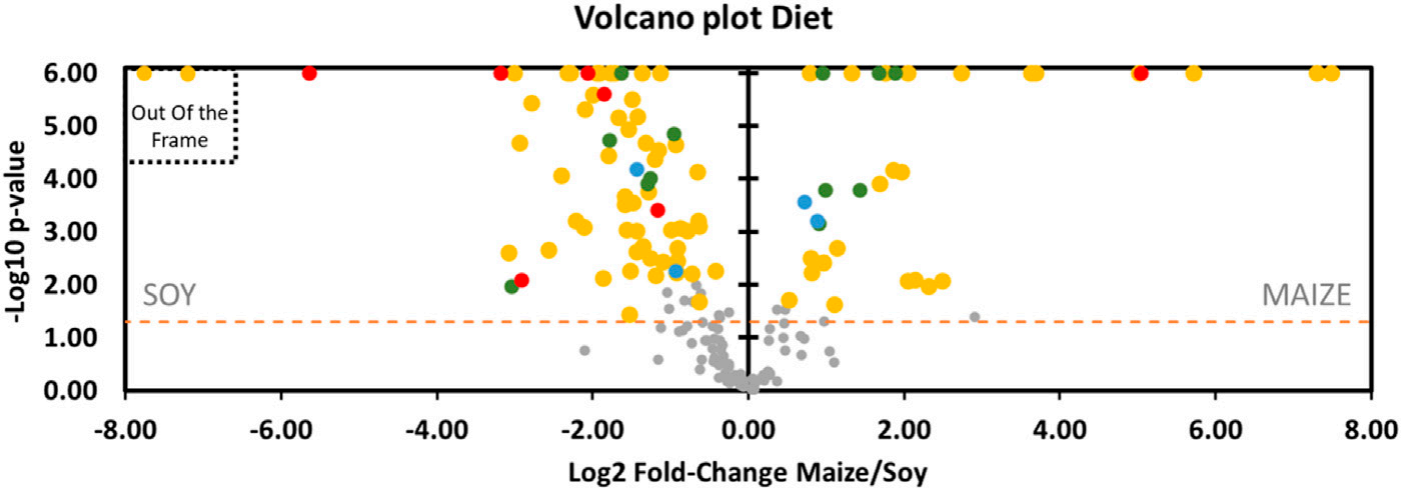
Volcano plot showing metabolomic data in rats (*n* = 57) according to the diet. Fecal metabolites over-expressed in rat fed maize are on the right and those fed with soy are on the left. The dashed line shows where *p* = 0.05 with points above the line having *p* < 0.05 and points below the line having *p* > 0.05, according to the 2-way ANOVA. Colors of the dots are in accordance with the Venn diagram. Red dots are for Diet*Pressure*Interaction, for example. Grey dots are not significant according to the 2-way ANOVA and the post-hoc testes. Please note, 2 yellow dots are out of the frame: Dipalmitoylglycerol (log2FC = −53) and Trans-3-hydroxycinnamic acid (log2FC = −51). Details are presented in Supplementary Data 1.

It can be observed on Graph 8 (Volcano Plot, Figure 8 + Supplementary Data 1 for the fold-change of metabolites) that the expression in the rat feces of a large part of the metabolites is favored by the soy diet (69 vs. 33) compared with the maize-based regime. In this scheme, 55 (vs. 24) of these compounds specifically linked to the diet (D) are over-expressed in the soy group. Four other metabolites undergo interaction effects (D*I) which are however not related to the effects of the pressure parameter directly. As the study focuses on dive, the details of these specific effects of the diet are lonely briefly exposed here.

The others metabolites are jointly influenced by pressure with either an additive (D*P, *n* = 12, green dots) or a synergistic effect (D*P*I, *n* = 7, red dots). From the point of view of the additive influence of the diet on pressure (D*P), six are more expressed with soy [(\xB1)-a-lipoic acid (fatty acid), methylthioadenosine, pterin, carnosine (dipeptide), L-gluamine (amino acid), L-leucine (amino acid)] while the others are more present with the maize diet [adenosine monophosphate (AMP), protoporphyrin, N-acetylgalactosamine (hexosamine), chenodeoxycholate (cholic acid), and two ionic types of n-acetylneuraminate (sialic acids)]. Among the D*P group, (\xB1)-a-lipoic acid, adenosine monophosphate, N-acetylgalactosamine and both type of n-acetylneuraminate are more presents after the dive. Concerning metabolites that underwent a synergic effect, it should be noted that 6 out of 7 D*P*Is (red dots, Figure 8) are to the left of the axis and that therefore this soy diet can have an important role in the expression of these metabolites depending on the pressure conditions. Among these 6, the thiopurine S-methylether is over-expressed in diving while the 5 other compounds [4-quinoline carboxylic acid, 7-methylguanine, arginine, Gly-Gly, thyrotropin releasing hormone (TRH)] are under-expressed (red dots, Figure 10). With the maize diet (red dot on the right side of Figure 8), pyrrole-2-carboxylate levels are decreased after diving (Figure 10).

These metabolites are also presented in Figure 10 showing the level of expression of the metabolites in rat stool as a function of exposure to pressure (control vs. diver).

**FIGURE 9:**
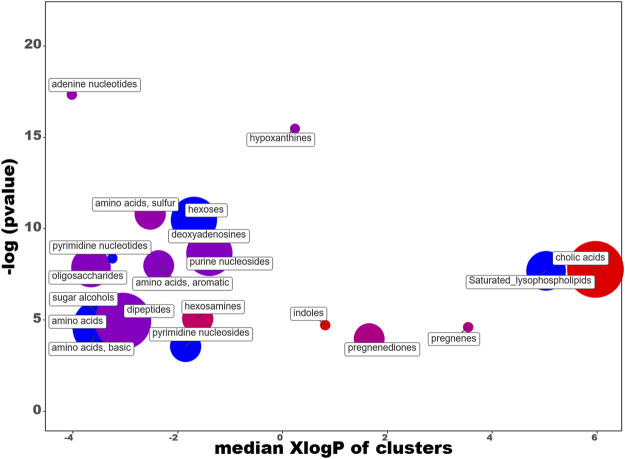
ChemRICH diagram according to the diet. Each disc reflects a significantly altered family of metabolites. These groups are developed from chemical similarities highlighted by a hierarchical Tanimoto map (not shown) accessible in the ChemRICH program. Enrichment *p*-values are given by the Kolmogorov–Smirnov test and displayed along the ordinate, with *p* values transformed in–log10. Disc sizes varies with the total number of metabolites. Red discs present groups of overexpressed metabolites in rats fed maize while blue ones show overexpressed compounds in rats fed soy. Purple color represents both increased and decreased metabolites. For example, more cholic acids are found in the feces of rats fed with maize compared to those fed with soy. Metabolite families are also scattered according to their increasing hydrophobicity (or decreasing hydrophilicity), from left to right along the abscissa (expressed as log P value).

In order to facilitate the analysis of our data, we have entered the differential expression rates in the ChemRICH database (Barupal and Fiehn, 2017) (www.chemrich.fiehnlab.ucdavis.edu), which allows access to the enrichment statistics. ChemRich plots together metabolites that are significantly altered according to their chemical similarity. 185 compounds accompanied by their variation factor and their *p*-values are uploaded to the ChemRICH server. 184 compounds are accepted. We analyze the effects of the diet and those linked to the dive. The ChemRich graph (Figure 9) based on diet shows a global overexpression (red circles) in the quantity of cholic acids (5 of the 8 identified compounds are altered), of indoles (altered ratio (AR): 0.7) and hesoxamines (AR: 0.8), in rats fed maize. Their key compounds are 5b-cholanic acid-3a-OL-7,12-DIONE, 5-hydroxyindole-3-acetic acid and galactosamine, respectively. In contrast, soy favors the presence of saturated lysophospholipides (AR:0.8), pyrimidine nucleosides (AR: 0.5) and pyrimidine nucleotides (AR: 0.7), basics amino acids (AR:0.6) and hexoses (AR:0.3). Not surprisingly, the levels of the different amino acids and dipeptides, or even sugars, vary depending on the diet. The changes in pregnenediones (AR: 0.5) and pregnene (AR:0.7) are more remarkable insofar as they affect cortexolone and 25-hydroxycholesterol, respectively. 25-Hydroxycholesterol that belongs to the D*I group is presented as an amplifier of inflammatory signaling (Gold et al., 2014). Concerning pregnenediones, the 2 (out of 4) metabolites analyzed are significantly altered. This group includes phytohormones (17a-Hydroxyprogesterone and cortexolone). Cortexolone, an inhibitor of testosterone receptors, is particularly overexpressed in maize diet with a fold-change of approximately 53 while 17a-Hydroxyprogesterone is overexpressed in soy diet (FD: 0.64).

**FIGURE 10:**
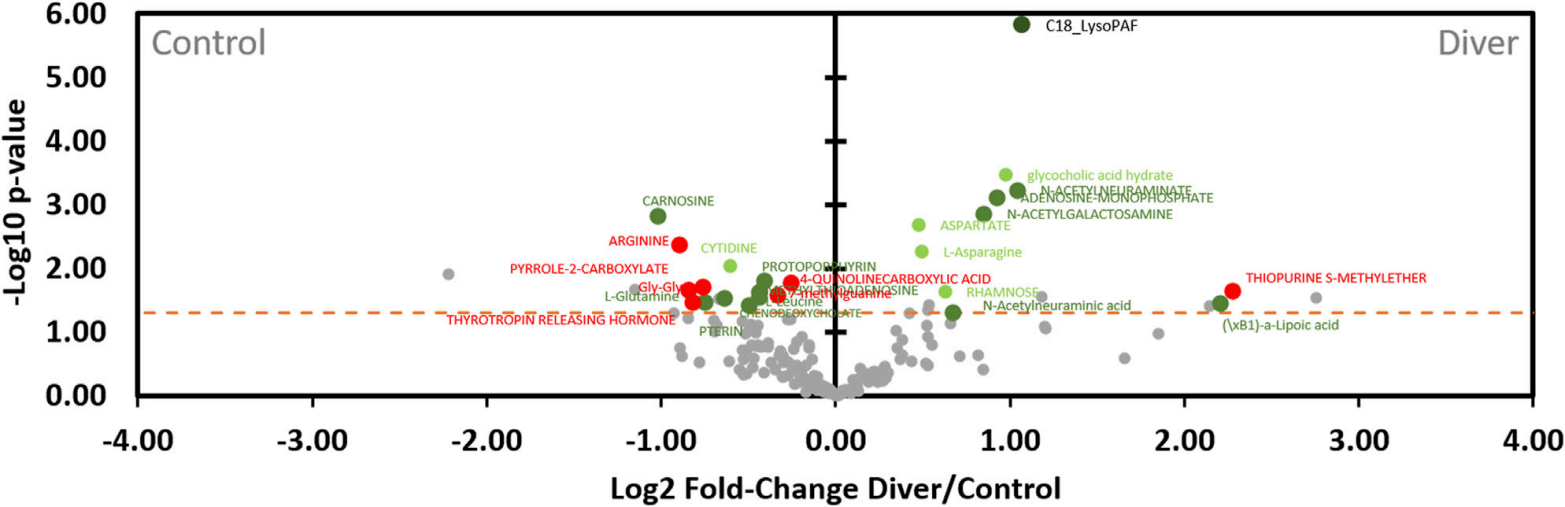
Volcano plot showing metabolomic data in rats (*n* = 56) according to the pressure exposure. Fecal metabolites over-expressed in control rats are on the left and those of diver rats are on the right. The dashed line shows where *p* = 0.05 with points above the line having *p* < 0.05 and points below the line having *p* > 0.05. Colors of the dots are in accordance with the Venn diagram and whose significance is true according to the 2-way ANOVA. Red dots are for Diet*Pressure*Interaction, for example. Grey dots are not significant according to the 2-way ANOVA and the post-hoc testes.

According to the volcano plot dedicated to the diving effects (Figure 10), levels of metabolites located on the right (*n* = 11) of the vertical axis are significantly (*p* < 0.05) higher in rats exposed to the hyperbaric protocol than those of control. In contrast, the quantities of metabolites on the left (*n* = 14) of the vertical axis are lower in divers’ rats compared to controls. In other words, the metabolites on the left are under-expressed and those on the right are over-expressed after diving, compared to the control rats. 25 compounds vary with pressurization, 6 of which (P and P*I) do not seem to be influenced by diets of this study. Regarding compounds lonely modified by the hyperbaric protocol (P, *n* = 5), diving induces a decrease in the levels of cytidine and an increase in the levels of aspartate, L-asparagine, glycocholic acid hydrate and rhamnose. An interaction effect greatly increases C18 LysoPAF after diving (P*I, *n* = 1). C18 LysoPAF has a very significant *p*-value. Among the D*P group, (\xB1)-a-lipoic acid, adenosine monophosphate, N-acetylgalactosamine and both type of n-acetylneuraminate are more present after the dive (*n* = 5). The first compound is favored by soy and others by maize. The other seven D*Ps are less present among divers, of which 5 (carnosine, L-glutamine and L-leucine, pterin, methylthioadenosine) are favored by soy and 2 by the maize diet (protoporphyrin and chenodeoxycholate). 6 out of 7 metabolites that underwent a synergic effect (D*P*I) are decreased in divers: arginine, pyrrole-2-carboxylate, Gly-Gly, thyrotropin releasing hormone (TRH), 7-methylguanine, 4-quinoline carboxylic acid are under-expressed and thiopurine S-methylether is over-expressed compared to the non-diver rats. As detailed above, their respective expressions are greatly influenced by the diet (Figure 8). Globally and compared to a maize diet, a soy-based diet increases their levels, except for the pyrrole-2-carboxylate.

**FIGURE 11:**
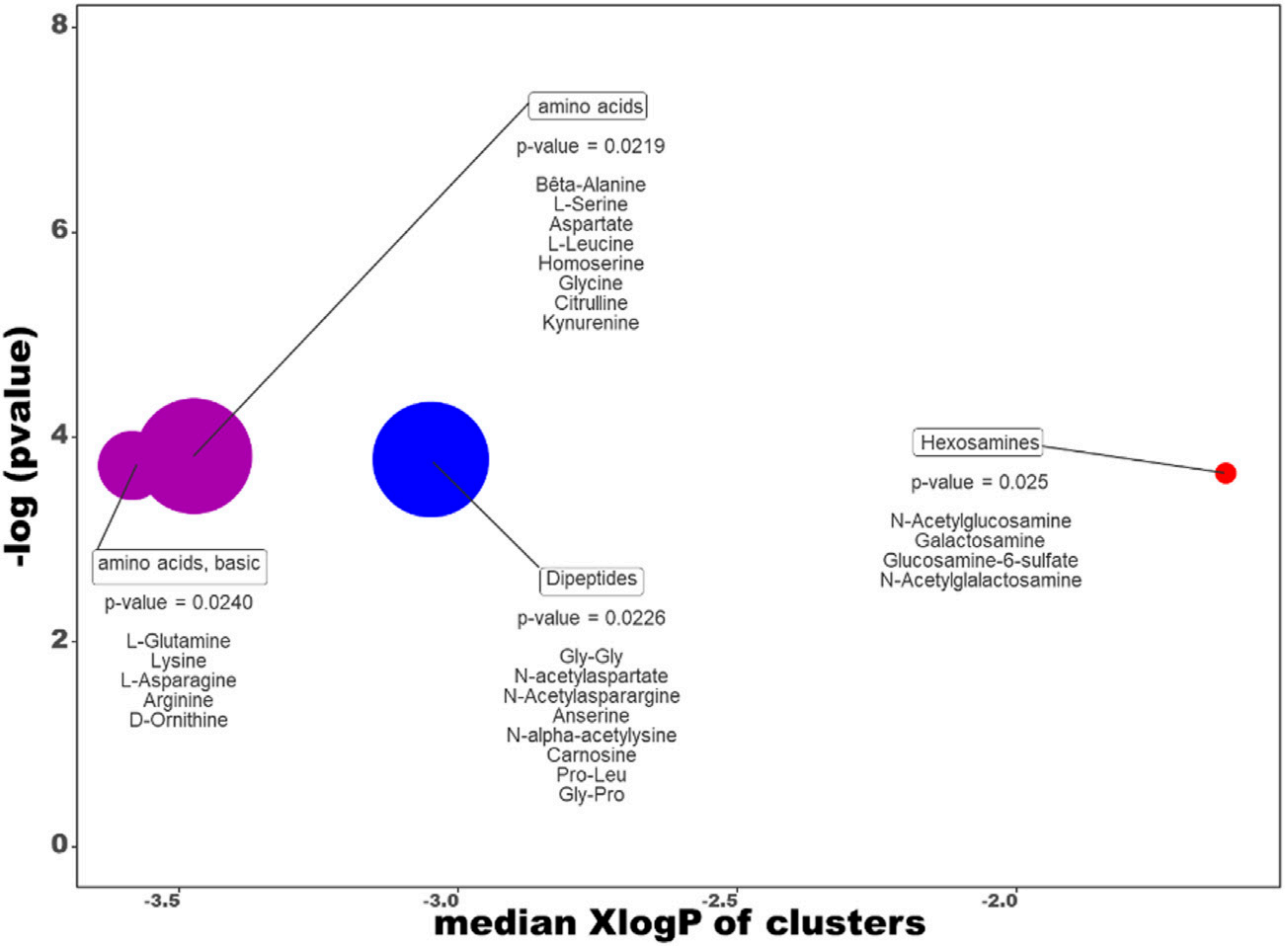
ChemRICH diagram according to the dive effect. Each disc reflects a significantly altered family of metabolites. These groups are developed from chemical similarities highlighted by a hierarchical Tanimoto map (not shown) accessible in the ChemRICH program. Enrichment *p*-values are given by the Kolmogorov–Smirnov test. Disc sizes varies with the total number of metabolites. Blue or red discs present groups of underexpressed or overexpressed metabolites in Diver compared to Ctrl rats, respectively. Purple color represents both increased and decreased metabolites. For example, there are less dipeptides in the feces of the diver rats.

The ChemRich graph (Figure 11 + supp data 1 for details) dedicated to the dive effect shows a global overexpression in resistant individuals compared to non-exposed rats (red circles) in the quantity of hexosamines (2/4 compounds are significant: N-acetylgalactosamine and glucosamine-6-sulfate).

In contrast, underexpression (blue disc) is visible in the feces of DCS-resistant animals for Dipeptides (4 decreased on 8 significantly altered, with N-acetyl galactosamine as key compound, other are gly-gly, anserine and carnosine).

In the clusters colored in purple, four amino acids (glutamine, arginine, leucine, kynurenine) are significantly decreased while two are overexpressed increased (asparagine and aspartate) in diver animals.

We have then set up and trained a random forest classification (machine learning) on whole data (Sampling: random with replacement; method: random input; number of trees: 300; Sample size: 35; Min Node: 2; Max depth: 20; Mtry: 4; Cp: 0.0001) in order to extract most contributing compounds for the description of diving effect. We have chosen the algorithm and the hyperparameters with the lowest error rate. The Out-Of-Bag (OOB), i.e., error rate, value of random forest is of 26.8%. The efficiency of the algorithm seems to be poor, according to the confusion matrix (73.2% of observed classes). For information, however, we have selected the top ten compounds, that is to say those having the greatest importance in the algorithm. The compounds selected in decreasing order of importance are: (\xB1)-a-Lipoic acid, C18 LysoPAF, Glucosamine-6-Sulfate, N-acetylneuraminate, Adenosine Mono Phosphate, N-acetylgalactosamine, 18-1 LysoPC, Phenylalanine, glycocholic acid hydrate, and mannitol. The 2 other algorithms (OBB of 30 and 34%) present different results depending on the settings of the hyperparameters, but four compounds are redundant. (\xB1)-a-Lipoic acid, C18 LysoPAF, Glucosamine-6-Sulfate, Adenosine Mono Phosphate have the greatest importance values according to the algorithm.

## Discussion

### Selection of Decompression Sickness-Resistance

This work makes possible the study physiological responses of DCS-resistance rat batches, and particularly their fecal metabolome from caecum, by comparing these asymptomatic Sprague Dawley rats exposed to the accident-causing protocol with the non-exposed ones. The main result of this analysis is a difference between the non-diver and DCS-resistant rats in their cecal composition.

As expected, diver rats selected here does not develop any of the clinical signs detectable by our tests. However, we do not deny the existence of subclinical signs or of pathophysiological responses to this hyperbaric protocol. In fact, we want to identify responses that are not pathological in these animals in order to better understand the pathological responses exposed in other studies (de Maistre et al., 2020; Vallee et al., 2021). And therefore, 31 diver rats which develop signs of DCS while they are exposed to the same protocol, are not analysis in this study but in another one (de Maistre et al., 2020) (Figure 1).

In addition to the clinical examination and the metabolomic data presented below, the blood analysis does not show any platelet or leukocyte mobilization or hematocrit variation following diving. Even lower levels of IL-1β, which is commonly presented for its pro-inflammatory properties (Stutz et al., 2009), are seen in these asymptomatic diving rats (compared to controls); this finding remains true despite the larger quantities in animals fed soy (legumes). For comparison with the previous study (de Maistre et al., 2020), IL-1β is only higher in soy-fed animals exhibiting symptoms of DCS. GPX activity is also lower in diving rat samples. It might therefore not have enough glutathione (tri-peptide formed from glutamate, cysteine and glycine) to oxidize in these rats, but on the other hand, we do not find a difference for plasma malondialdehyde (Tbars), the oxidative degradation product of unsaturated fatty acids (lipids). A decrease in GPX activity might also be linked to a containment of TBARS. A response to oxidative stress remains to be considered in these asymptomatic rats despite a quasi-non-inflammatory state. To be complete, it is to be noted in the previous study (de Maistre et al., 2020), TBARS levels are more important in DCS rats, compared to NoDCS rats.

### Influence of the Diet on Blood Analysis, Exhaled Hydrogen and Cecal Content

The topic of this work is not intended to analyze exhaustively the influence of a diet on the different variables, but to identify those particularly influenced by these two diets in order to better extract those which vary following a dive. This study focuses on the fecal metabolome of two batches of rats and we want to benefit from a contrast effect by modulating the diet. So, we deliberately briefly discuss the effects linked to food.

Soy and maize diets are previously described as factor greatly shaping the metabolomic profile (de Maistre et al., 2020). Our results emphasize that diet cause great variation in metabolite levels. Our AHC analysis also confirms differences between soy and maize diet in the non-exposed and the DCS-resistant animals. In addition to the metabolomic analysis, we firstly use the measurement of exhaled hydrogen before the hyperbaric exposure. Thus, the impact of diet on the activity of the gut microbial community is indirectly appreciated with a more important averaged exhalation of H2 in rats fed soy (vegetable), which can be interpreted as a greater intestinal fermentation (Bernalier-Donadille, 2004; Louis and Flint, 2017). A greater mobilization of certain bacterial strains might be suggested.

We are able to determine that diet influences initial blood parameters Thus, the SOY rats have more platelets but their mobilization does not seem to be more affected. It is also noted that the volume of their erythrocytes is lower. Amongst the causes of microcytosis are inflammation (see below) and iron deficiencies (Blann and Ahmed, 2014), but soy is reputed to be richer in iron, compared to maize (Leng et al., 2019). Amongst other things, this might also indicate that erythrocytes are older (Blann and Ahmed, 2014) or that their pool is slowly renewed. However, the volume is not changed after the protocol. In addition, nor a small MCV nor this difference in platelet counts seem to particularly promote or impede a safe dive.

The measurement of plasmatic IL-1β is higher in the SOY rats, which indicates the existence of a more important inflammation state (not necessarily inflammation) in this batch, while their clinical status is not called into question. Compared to Divers rats, a higher level of IL-1β is also true in Ctrl Rats.

As far as the fecal metabolome is concerned, as waited its composition depends on diet. So, 102 out of the 185 metabolites identified vary as a function of diet (Supplementary Data 1).

A few metabolites are getting attention because different diets are regularly linked to different organizations of the activity of the intestinal community. This usually involves the decrease or proliferation of bacterial populations (modulation in phosphorylcholine amount), which try to adapt to the new diet, while the host tries to contain it through inflammatory processes (Maslennikov et al., 2019). As the diet is the same for control rats and divers rats during at least 2 weeks before the sampling of fecal content, it is likely that any changes in the cecal composition come from a reorganization of the activity of the intestinal microbiota community.

In the MAIZE rats, such a reorganization of the microbiota, in the way of a greater diversity, is suggested by the higher amounts of hippurate (FD = 157) (Swann et al., 2011; Bajaj et al., 2014; Barbara et al., 2017; Pallister et al., 2017) and 2,6 quinolinediol (FD = 178). Hippurate is also described as a representant of *bacteroides*, one of the most important group of anaerobic Gram-negative bacilli in the intestine (Bajaj et al., 2014). 2,6 quinolinediol has antibiotic properties and it is thought to control an explosive development of the microbial community in the intestine (Walker et al., 2014). The phytohormone cortexolone, a derivative of cortisol (Doden and Ridlon, 2021) and also a testosterone receptor inhibitor (Korach-Rechtman et al., 2019), is also over-expressed in this group (FD = 52). Especially, the phytohormone cortexolone which is specific to the gut microbial community, with a high prevalence in maize fed rats, has potent effect on the energy metabolism of sugar, such as glucose-6-phosphate. This point is previously presented in another work concerning a selected strain of rats resistant to decompression sickness with lower levels of cortexolone and glucose-6-phosphate (Vallee et al., 2021).

Conversely in the SOY rats, it would seem that the regulation mechanism differs where there are higher levels of the cecal phosphorylcholine (FD = 0.12) and plasmatic IL-1β. An excess of phosphorylcholine (choline), a molecule which in vaccination strategies (Trolle et al., 2000; Ohori et al., 2020), is associated to an activation of the immune system. Phosphorylcholine is contained in all Gram-positive and Gram-negative bacteria, and its detection by the M cells (microfold cells) of the cecum, during bacterial over-representation or when there is a digestive tract lesion allowing infiltration, might participate in the inflammatory syndrome. More globally, the over-expression of this choline suggests that the bacterial populations have been reshuffled.

Finally, it seems that these diet-induced reshuffles are not necessarily detrimental for these selected animals while they involve metabolites with potent biological effects.

The previous analysis (de Maistre et al., 2020) comparing uninjured diving animals to DCS animals determined that 103 metabolites, vs. 102 in this study, varied with these same diets. One would expect these compounds to be the same, but 27 compounds are different (Supplementary Data 2). Finally, 116 compounds can be retained as being influenced by the diet if we chose to retain them all. If we are more restrictive, we can consider that 89 compounds are really influenced by the diet, considering that the remaining 27 metabolites are questionable. These 27 metabolites may also be attributed an indirect role in DCS. This point is decisive because it allows to appreciate one of the limits of such a study. Typically, among the compounds believed to vary depending on the diet and which are only found in this study, L-leucine (D*P), chenodeoxycholate (D*P) and arginine (D*P*I) are in the interaction zones of the Venn diagram. Their interpretation therefore calls for caution. However, it goes without saying that these metabolites cannot exist without food, and that by nature they are therefore influenced by diet and also by microbial activity. We must remember that the aim of this study is essentially to understand better the effect of diving. Hence, the use of two different diets is a mean to spotlight, by contrast effect, metabolites possibly linked to a safe dive. Finally, this method allows us to restrict our analysis to 25 metabolites influenced by the pressure exposure (Venn Diagram of the Figure 7).

### Metabolome of Uninjured Rats After a Provocative Dive

This study highlights that rats exposed to this dive protocol have a different fecal metabolome of the caecum regardless of the diet, even if the affected metabolomic fingerprint takes into account the initial food intake. A dichotomy between the pressure groups on this heatmap, or PCA, is clearly not as noticeable as in the previous studies (Vallee et al., 2021; de Maistre et al., 2020). Nonetheless, we can establish a link between the expression of 25 fecal metabolites and the non-pathological response to the dive (Venn Diagram of the Figure 7). Obviously, diet *per se* is not enough to protect from DCS but this result has to be compared to the 37 metabolites detected in the study dedicated to the DCS (de Maistre et al., 2020) (Supplementary Data 2), or to the 81 metabolites identified in a strain of rats resistant to decompression sickness (Vallee et al., 2021).

It is interesting to note that very few metabolites are in common with the two previous studies (de Maistre et al., 2020; Vallee et al., 2021). And when they are, they are mainly related to the effects of diets, and not necessarily to the declaration of a DCS. Nevertheless, carnosine (D*P) and leucine (D*P) are here related to the pressure exposure group and also to the possibility of declaring a DCS (de Maistre et al., 2020) (Supplementary Data 2). Carnosine quantities are reduced in the divers of this study, and they are even more in the injured rats. As for the amounts of leucine, they are here reduced by the effect of pressure, but they are increased in the event of proven DCS. Other compounds from this study are also selected over generations of rats bred to resist DCS (Vallee et al., 2021). Thus, decreases in chenodeoxycholate and glucose-6-phosphate are here linked to the effect of pressure, but also during the selection of resistant rats by breeding.

Again, we must also remember that (\xB1)-a-Lipoic acid, C18lysoPAF, Glucosamine-6-Sulfate, Adenosine Mono Phosphate are redundant metabolites according to the statistical analyses (ANOVA2, random forest classification, ChemRich) and that the discussion will take this into account.

### Expression of Metabolites Linked to Energy Expenditure

Changes in the amounts of some metabolites portend a change in the energetic allowance at the gut level in diving rats. Concomitantly, we record a new shape in hormonal profile including decrease in Thyrotropin Releasing Hormone (TRH) in diving rats.

In mitochondria, the glycerol-3-phosphate (I) shuttle is a mechanism that regenerates NAD+ from NADH, a by-product of glycolysis. In the maize-fed rats, glycerol-3-phosphate levels are lower in divers compared to controls, while the reverse is true in the soy-fed rats. One might expect the same profile for NAD+ but the only significant difference concerns much lower NAD+ levels (D) in maize-fed rats. On another hand, glucose 6-phosphate (I) has the same distribution profile as glycerol-3-phosphate. Only glucose 6-phosphate among I metabolites of this study is presented to be linked to the diet in the list of the previous study (de Maistre et al., 2020). It is decreased in strain of rats resistant to DCS (Vallee et al., 2021). Glucose 6-phosphate is a primary metabolite involved in energy metabolism. It lies at the start of two metabolic pathways: glycolysis and the pentose phosphate pathway. The pentose phosphate pathway generates NADPH and ribose-5-phosphate from glucose-6-Phosphate and NADP+. Actually, ribose-5-phosphate belongs to D*I group of the Venn diagram, and it follows a pattern similar to that of glucose-6-phosphate: in the maize-fed rats, ribose-5-phosphate levels are lower in divers compared to controls, while it remains unchanged (or very tiny increase) in soy-fed rats. Unfortunately, neither NADP+/nor NADPH are measured in this study, but an older work from Dodd and Faiman (1978) provides information approaching. They conclude oxidation of NADPH occurs as soon as animals are exposed to hyperbaric oxygen because they have observed that hyperbaric oxygen rapidly increases both cortical NADP+ and the NADP+/NADPH ratio, and drastically decreases NADPH at all stages of oxygen exposure (Dodd and Faiman, 1978). They reach the pressure of 6100 mbar of 100% oxygen for 16 min maximum (Dodd and Faiman, 1978) while our study reaches 2100 mbar of PiO2 for a much longer duration (45 min at the top stage). Underproduction or degradation of NADPH, necessary for the formation of glutathione, strongly reduces the cellular capacities to fight against oxidative stress. We therefore register lower levels of plasmatic GPX activity in Diver, and it indicates lower glutathione levels, hyperbaric oxygen diffusing throughout the body. NADPH is also required in the neutrophile activation (Welch et al., 2009). Once more, no mobilization of platelet or leukocytes is noticed in this study. This does not explain the opposite behaviors of SOY and MAIZE batches for glucose-6-phosphate or glycerol-3-phosphate, unless an essential metabolite provided by one of these diets interferes in chemical reactions. In a purely hypothetical way, this actor might just as well be the 25-Hydroxycholesterol (D*I) that is touted as an amplifier of inflammatory signaling (Gold et al., 2014), as any other metabolite with a potent effect as can be hormones (please see ChemRich analysis).

The consequence of a decrease in the hypothalamic hormone Thyrotropin Releasing Hormone (TRH) acting on the adenohypophysis (*via* TSH) and the synthesis of thyroid hormones [thyroxin (T3) and triiodothyronine (T4)] may also be substantial (Šošić-Jurjević et al., 2014). Nonetheless, TRH differences are not associated to DCS occurrence but lonely to a soy-induced effect in previous study (de Maistre et al., 2020). In this work, TRH (D*P*I) seems almost undetectable in the MAIZE group, but its levels are lower in SOY Divers compared to the SOY Controls. We therefore suggest that soy increases cecal TRH level while diving decreases it. The overriding question is whether this conclusion is also a reality in the rest of the organism and what would be the consequences over a short to moderate period of time? Interestingly, the amount of transthyretin, a blood carrier for T3 and T4, is reported to be decreased in diving rats (Lautridou et al., 2016), and level of transthyretin might be linked to those of T3 and T4 (Episkopou et al., 1993; Sintzel et al., 2004; Morgado et al., 2007; Park et al., 2019).

### Expression of Metabolites Linked to Biliary Acids

Bile acids are the products of cholesterol catabolism. Gut microbiota participates in the biotransformation of bile acids which can affect the bioavailability of lipophilic compounds (Zhang et al., 2020). Bile acid modifications may affect poorly water-soluble compound transports. The gut microbiota can also affect the bioavailability of oral drugs by modulating the metabolism of bile acids (Zhang et al., 2020). Discoveries have shifted currently accepted definition of biliary acids to signaling hormones endowed with a wide array of endocrine functions with specific nuclear and membrane receptors, including G-protein-coupled receptor associated with the intracellular accumulation of cAMP (Sato et al., 2008). The wide array of endocrine functions of this biliary acid comprises decreases proinflammatory cytokine production by macrophages (Kawamata et al., 2003), or enhances energy expenditure by promoting intracellular thyroid hormone activation (Watanabe et al., 2006). Cholic acid (CA) and chenodeoxycholic acid (CDCA) are primary bile acids which are synthesized in hepatocytes from cholesterol. Conjugated primary bile acids, such as glycocholic acid hydrate (GCAH), can be biotransformed into secondary bile acids by the gut microbiota in the intestine, forming deoxycholic acid (DCA) from CA and lithocholic acid (LCA) from CDCA. Lithocholic acid can be metabolized into dehydrolithocholic acid (5b-cholanic acid-3-one or DHLCA) (Yoshiki et al., 1985). Biliary salts are essentially deconjugated in gastrointestinal tract by strict anaerobic bacteria (Gilliland and Speck, 1977). Chenodeoxycholate is decreased over generations of rats bred to resist DCS (Vallee et al., 2021). In this study, chenodeoxycholate is linked to diet and pressure groups. Its amount is lower in diver rats, even though maize-fed rats have more. An increase of LCA is also linked to maize but no pressure effect is noted, and then, dehydrolithocholic acid (I) levels are the same in divers compared to controls. Intriguingly, DHLCA decreases in the divers of the soy-fed rats. We can assume, without conviction, that the decrease in CDCA displayed in diver group does not seem to have any consequence on the formation of LCA. However, glycocholic acid hydrate (P) the primary bile salt is enhanced (FD = 1.97) in all divers, what suggests a decrease in deconjugation. On the other side, a significant decrease in LCA is noted in DCS rats compared to uninjured rats in the previous study, with a diet effect that persists (Supplementary Data 2), while the glycholic acid hydrate levels remain similar (de Maistre et al., 2020). This suggests a disturbance in the liver due to the pressure initially, then to an alteration of the bacterial intestinal strains responsible for the production of bile acids or to a change in intestinal absorption secondly. Intriguingly, the action appears to be specific for CDCA and LCA: neither CA nor DCA seem to be impacted by the dive or the DCS (Supplementary Data 2) (de Maistre et al., 2020). Even more disturbingly, only males of rats selected to resist DCS (but not exposed to provocative dive) have decreased CDCA and DCA levels, with no changes in LCA, CA, and GCAH (Vallee et al., 2021). Considering 5b-cholanic acid-3-one, it has only been mentioned in our previous study when it might have deserved more attention: it is over-expressed in female rats while it is known that sex is an influencing factor in DCS (Vallee et al., 2021).

### Expression of Metabolites Linked to Structural Properties

#### Nucleosides

4-quinoline carboxylic acid (D*P*I) levels are slightly lower in SOY Divers compared to SOY Ctrl, and they are not impacted in maize-fed rats. 4-quinoline carboxylic acid has antiviral activity because it inhibits the pyrimidine biosynthesis *via* the mitochondrial enzyme dihydroorotate dehydrogenase (DHODH). Pyrimidines are essential for the biosynthesis of DNA, RNA, and phospholipids. Inhibition of DHODH causes reduced levels of pyrimidine nucleotides that in turn trigger various activities such as anticancer, immunosuppressive, antimalarial, and antifungal. Additionally, inhibition of DHODH can be beneficial for spinal cord injury and rheumatoid arthritis (Das et al., 2013). One might suggest that soy increases cecal 4-quinoline carboxylic acid level, while diving decreases it, that is to say that inhibition of pyrimidine biosynthesis may be lifted, or pyrimidine amount, and its derivative products (purine are compounds made up of a pyrimidine ring fused to an imidazole ring), may be increased in soy-fed rats. Thiopurine S-methylether is over-expressed in soy-fed rats but, nor cytidine (P) a pyrimidine nucleoside nor 7-methylguanine (D*P*I) a purine nucleoside are greater in soy-fed rats after the dive; on the contrary, their amounts are lower.

In parallel, fecal adenosine-monophosphate (AMP or 5′AMP) levels are greater in the Diver rats (FD = 1.90). AMP is a nucleotide. Along with uridine monophosphate (UMP), guanosine monophosphate (GMP) and cytidine monophosphate (CMP), AMP is one of the monomers that make up RNA, and its increase might come from degradation. AMP is also described as a neutrophil-derived paracrine factor that elicits chloride secretion from intestinal epithelial cell monolayers. In intestinal inflammation, its production may contribute to the secretory diarrhea that occurs in states characterized by neutrophil migration across the crypt lumen (Madara et al., 1993). In this study, such a greater level of AMP might be linked to an inflammatory response. In the previous study (de Maistre et al., 2020), AMP was associated to diet while it is linked to diet and pressure effects in this one (Supplementary Data 2).

Deoxyguanosine Monophosphate (dGMP) (I) is a deoxyribonucleotide of DNA. In the maize-fed rats, dGMP levels are lower in divers compared to controls, while the reverse is true in the soy-fed rats. dGMP has structural properties and also regulatory functioning G-quadruplex structure per exemple (Wang et al., 2020)^,^ (Curtis and Liu, 2013), with a potent regulation role in gene promoting. Intriguingly, higher extracellular concentration of dGMP is also associated to an enhanced oxidative stress resistance in an extremophile bacteria (Li et al., 2013). But we must recall free extracellular DNA provides nutrition to bacteria, and changes in concentration might be linked to the activity of the microbial community. Bacteria cell releases cytoplasmic contents including DNA components into the microenvironment when death or growth processing (Li et al., 2013). Such difference might obviously be thought in the spirit of previous paragraph where hormonal or energy strategy reshuffles may be suspected.

### Amino Acids and Dipeptides

Beyond their role as residues in proteins, amino acids or dipeptides participate in a number of biological processes. It is difficult to say whether their under-representation implies under-production, over-degradation or over-absorption, or any other process that alters stocks, but we can at least give them the role of constituting the living.

The availability of amino acids glutamine and leucine, as arginine (D*P*I), is decreased in divers, while aspartate and asparagine level are enhanced. For instance, glutamine and aspartate are precursors of nucleotides and arginine is a precursor of nitric oxide but not only. Pyrrole-2-carboxylate is included in the metabolism of the L-proline (Heacock and Adams, 1975) itself synthesized from arginine (D*P*I) and glutamine/glutamate. Pyrrole-2-carboxylate (D*P*I) levels are lower in MAIZE Divers compared to MAIZE Control group. L-proline is not measured in this study. Generally, a decrease in pyrrole-2-carboxylate has a weak significant physiological action (Pérez ‐ Arellano et al., 2010; Wu et al., 2011).

The quantities of Gly-Gly are two less important in soy-fed rats who dive, while they have not different in those fed maïze. The other dipeptide (alanine and histidine) carnosine (D*P) is associated to diet and pressure effects in this study and also to diet and the clinical status after the provocative dive in the previous study (de Maistre et al., 2020). It is more abundant in soy-fed rats in both studies. Its quantities are lower in the divers of this study, and they are even more in the injured rats (de Maistre et al., 2020). Because of its biological properties, carnosine is also presented in the section dealing with oxidative stress.

### Cell Wall

N-acetyl galactosamine is associated to diet and pressure effects in this study and also to diet despite a provocative dive in the previous study. Exposure to pressure is linked to greater amount. Amounts are higher in maize-fed rats. On another side, galactosamine (D) amounts are higher in soy-fed rats. N-acetyl galactosamine links carbohydrate chains in mammalian mucins (Carraway and Hull, 1991). It is also an element of lipopolysaccharide displayed on bacterial cell wall (Bernatchez et al., 2005; Leyn et al., 2013). Additionally, it serves as substrate in N-acetyl β-galactosidation, a type of post-translational modification of protein in organisms, including bacterial pathogens (Abu-Qarn et al., 2008). Given the multiple roles played by galactosamine/N-acetyl galactosamine metabolism, it is also proposed to be considered as a bacterial virulence index (Zhang et al., 2015). Finally, any change in N-acetyl galactosamine concentrations can be perceived as resulting from bacterial destruction or alteration, or from a new allocation of resources which increases the overall amount of N-acetyl galactosamine. However, neither galactosamine, nor galactose, or yet another amino sugar seems to be particularly affected by diving. The interpretation is rather in favor of a slight reshuffle linked to hyperbaric conditions as long as the diet is stable.

N-acetyl neuraminate, also known as Neu5Ac, has the same profile as N-acetyl galactosamine. It is associated to diet and pressure effects in this study and also to diet despite a provocative dive in the previous study. It is over-expressed in diver rats. This sugar (sialic acid) is involved in many biological and pathological phenomena because it occupies the terminal positions in numerous macromolecules, such as the glycans of glycoproteins, with a broad impact on gut microbiome structure, such as in inhibiting autoimmune signaling in the gut (Coker et al., 2021). N-acetyl neuraminate is a compound useful in the composition of biofilms sometimes necessary for the sporulation of certain bacteria entering dormancy following environmental stresses (such as temperature, high iron, oxygen concentration, hydrodynamic effects and food matrix composition or starvation) (Mohsin et al., 2021). N-acetyl neuraminate can be made from N-acetyl glucosamine (Gao et al., 2011). The quantities of the latter are similar whatever the group considered in this study.

Rhamnose (P) is a component of the outer cell membrane of acid-fast bacteria in the *Mycobacterium* genus, which includes the organism that causes tuberculosis (Golan et al., 2011). It is over-expressed in rats exposed to pressure.

The increase in these three metabolites entering into the composition of the walls suggests cell alteration due to exposure to the diving protocol. However, it should be noted that we do not find any significant difference in the rates of elements essential to the walls, such as the excess of phosphorylcholine found in DCS animals (de Maistre et al., 2020). Also, this result deserves to be explored.

### Expression of Metabolites Linked to Oxidative Stress

Oxidative stress is regularly evoked in diving (Thom, 2009; Morabito et al., 2011; Eftedal et al., 2013; Wang et al., 2015; Blatteau et al., 2018). We have mentioned above lower GPX activity in blood of diver rats that might be linked to oxidative stress. The dipeptide carnosine (D*P) is briefly presented in the section dealing with amino acids. In cecum, its quantities are lower in the divers of this study, and they are even more in the injured rats (de Maistre et al., 2020). In recent years, small peptides including carnosine are presented as quasi-hormones and pharmaceutical agents that can be absorbed from the intestine and modulate the cells’ physiological functions (Vahdatpour et al., 2019). Like glutathione (peptide B) the substrate of GPX, carnosine (also known as peptide A) has anti-oxidant activity and anti-inflammatory effects (Boldyrev et al., 2013; Caruso et al., 2019). And because we now know that it has a broad spectrum of action (Caruso et al., 2020), the role of carnosine in the intestinal microbiota is emerging (Han et al., 2021). It improves mitochondrial function, glucose metabolism (Tsoi et al., 2011), albumineria and hyperuricaemia (Boldyrev et al., 2013; Caruso et al., 2019; Caruso et al., 2020) and others effects on depression, cardiovascular diseases (Tsoi et al., 2011; Caruso et al., 2020) and thermoregulation (Nagai et al., 2012) involving the pituitary axis are questioned. Blood thyroid hormones are also modified by carnosine supplementation (Bao et al., 2015). We suggest a decrease in carnosine may be linked to a decrease in antioxidant capacity, that reflects an increased oxidative process due to dive. Disturbingly, anserine, a methylated derivative of carnosine with similar properties (Katakura et al., 2017), is very strongly expressed in the feces of injured rats (de Maistre et al., 2020) but it is not in this study that considers uninjured animals. Also interestingly, carnosine is associated to (\xB1)-a-Lipoic acid (ALA) (please, see below), a metabolite presented in some studies dealing with inflammatory process (Bao et al., 2015).

Protoporphyrin is associated to diet and pressure effects in this study and to diet after the provocative dive in the previous study (de Maistre et al., 2020). Amounts are higher in soy fed rats. Divers have lower levels of protoporphyrin while no difference is observed in previous study between NoDCS and DCS rats (Supplementary Data 2) (de Maistre et al., 2020). Protoporphyrin or metal-free protoporphyrin correspond to a precursor type of ferrous-protoporphyrin (heme) or zinc-protoporphyrin which will result in biliverdin and bilirubin (Labbé et al., 1999). In blood, protoporphyrin with a chelated metal such as iron evidently is bound to a heme site on globin. In gut, it could originate from shedding of cells or bleeding, and its amount excreted will depend on the balance between the amount delivered to the lumen and its loss from the lumen (Young et al., 1990). Depending on aerobic conditions, intestinal bacteria convert a fraction of the heme to protoporphyrin or another derivative (Young et al., 1990). Suppression of gut anaerobic bacteria decreases protoporphyrin production or fundamentally changes the type of porphyrin production (Beukeveld et al., 1987). Under unspecified but probably aerobic conditions *in vitro*, it does not appear that fecal bacteria are capable of splitting open the porphyrin ring to produce non-porphyrin derivatives of heme (Beukeveld et al., 1987). The clinical importance of protoporphyrin is not limited to iron, however, its amount can influence heme catabolism, leading to modulation of oxygen transport and bilirubin and CO production. Metal-protoporphyrin is taken up into bacterial cells where it perform essential cellular pathways, and a decrease may disrupts the respiratory chain, and induces reactive oxygen species that are toxic to bacteria (Stojiljkovic et al., 2001). Because of the high levels of oxygen (max inhaled PiO2: 2000 mbars) caused by the hyperbaric protocol and the lower expression of protoporphyrin in diver rats, we fear that diving may be detrimental to gut bacteria, more specifically anaerobic ones. In the strain of rats bred to resist against DCS, biliverdin is under-expressed in feces (Vallee et al., 2021).

Pterin (D*P) is under-expressed in diver rats whatever is the diet. Amounts are higher in soy-fed rats. Pterins are product derived from folic acid. They are widely conserved biomolecules that have probiotic and pathogenic roles played by gut bacteria. It has antioxidant and anti-inflammatory properties (Feirer and Fuqua, 2017; Park et al., 2020).

### Expression of Metabolites Linked to Inflammatory Process

Rats exposed to the hyperbaric protocol have a metabolome fingerprint that slightly differs from control group, involving energetic and hormonal regulation compounds and also some related to oxidative stress, whether in the host or in the microbial community.

Protoporphyrin pterin and carnosine are mentioned in previous paragraphs as been probably linked to oxidative stress while AMP may be linked to an inflammatory response in diving.

(\xB1)-a-Lipoic acid (ALA) levels are about five times higher among divers compared to controls of maize-fed rats, while it increases a little less than double in the soy-fed rats. ALA can modifies the epigenetic regulation of DNA structure at the histone proteins level (Bassett and Barnett, 2014) and has antigenotoxic effect H_2_O_2_-induced (Mottola et al., 2021). ALA has antioxidant activity and anti-inflammation properties (Rochette et al., 2013; Bao et al., 2015; Moos et al., 2016), because of its role in the glutathione reaction (Rochette et al., 2013). It is an essential cofactor for redox reaction, and therefore for mitochondria (Mayr et al., 2014). ALA has also a role in microbial metabolism (Ullah et al., 2016). We suggest ALA participates to alleviate oxidative stress of the uninjured animal, but it is not essential in DCS prevention (Supplementary Data 2) (de Maistre et al., 2020).

The levels of C18_LysoPAF, that undergoes interactions (P*I), are twice as high in divers (FD = 2.09). C18_lysoPAF may have direct or indirect inflammatory effects (Dyer et al., 2010), but it is essentially a precursor of PAF, the Platelet Activation Factor that can be released by enterobacteria such as *E. coli* and which is a potent mediator of inflammatory diseases and septic shock or gastric ulceration (Denizot et al., 1989). Its analog C_16 has similar properties with a broad spectrum of inflammatory actions, at the level of IL-1β or on the glutathione ratio per example (Riaz et al., 2018; Riaz et al., 2020). Considering literature (Lee and Polin, 2003; Welch et al., 2009; Dyer et al., 2010; Riaz et al., 2020), this result is of prime importance because it suggests that the modulation of the quantities of PAF is a non-negligible possibility after an hyperbaric exposure, including dive. Unfortunately, we do not measure PAF in this study, and the increased C18_LysoPAF can be read in many ways. As no mobilization of platelet or leukocytes is noticed in any batch, an interpretation similar to Welch’s work, that is to say in the way of an anti-inflammatory effect of LysoPAF, might be satisfactory: this team demonstrates LysoPAF dose-dependently inhibits neutrophil NADPH oxidase activation through an increase of intracellular cAMP levels, a mechanism which is also believed to be effective in inhibiting platelet aggregation (Welch et al., 2009). Actually, we notice lower plasmatic levels of IL-1β and GPX activity, which may be attributed to a lower inflammatory status managed by a response to an oxidative stress, in diving rats.

Among the metabolites which undergo a synergistic evolution (D*P*I), but which do not evolve in animals-fed maize, Thiopurine S-methylether is interesting. Thiopurine S-methylether levels are higher in SOY Divers compared to SOY Ctrl, and there is no difference between maize-fed rats. Actually, it seems almost undetectable in the MAIZE group. Thiopurine S-methylether, also known as 6-Methylmercaptopurine or 6-mercaptopurine (6-MP) exists in all living organisms, ranging from bacteria to humans. The anti-inflammatory activity of 6-MP might target not only human macrophages, epithelial cells, and lymphocytes, but also the gut microbiota, by impacting either growth or physiological conditions like motility (or both) of bacteria linked to Crown Disease (Migliore et al., 2018). 6-MP impairs the ability of *E. coli* to adhere to, and consequently to invade, human epithelial cells. Notably, phagocytosis of *E. coli* strains treated with 6-MP by human macrophages is also reduced, suggesting that 6-MP affects *E. coli* cell surface determinants involved both in interaction with epithelial cells and in uptake by macrophages (Migliore et al., 2018). 6-MP is able to target pathobiont virulence, possibly *via* its ability to inhibit *de novo* purine biosynthesis (Ha et al., 1990; Antoniani et al., 2013). Notably, levels of the methylated purine nucleoside 7-methylguanine are lower in Soy Divers, compared to Soy Ctrl. It therefore suggests, in fed-soy rats, the increase in 6-MP could be linked to the decrease of cecal 7-methylguanine level. In fed maize rats, 6-MP as 7-methylguanine levels may not be affected due to their very low amount. However, the methythioadenosine (D*P), a purine derivative balances this suggestion insofar as its lower expression in diver groups is true in soy fed rats but also in those fed corn. These proposals are essentially hypothetical insofar as each metabolite deserves a specific study, but they nevertheless denote a different shape between the control group and the divers.

### Moderation

Like any model, our experiment intrinsically has limits in terms of choice of species, intra or inter-individual variability that a control group can only imperfectly erase.

This work obviously has not analyzed all the metabolites composing the faeces, in terms of pure technical analysis or in terms of discussion of each of the compounds identified. It is therefore possible these missing metabolites may play a determinant role.

We write that a certain number of cecal compounds are linked to the pressure, hyperbaric exposure or diving effect and that may also may be link to maize or soy diet. However, as this work study fecal metabolome, it is not possible to exclude the fact that another diet can have an influence.

We assume that the diets of animals differ, and that a 30-days adaptation period may not seem short regarding the microbiote community. This could induce inflammation and thus make the animals more susceptible to the hyperbaric exposure effect (de Maistre et al., 2020). However, this does not seem sufficient to explain differences between the control and the diving group in this study. At least, we have not demonstrated a difference in susceptibility to DCS in rats receiving those two different diets (de Maistre et al., 2020).

We assume that the batch selection involves that Ctrl are slightly heavier (+7%) than divers and, in a study dealing with diving such a difference could be perceived as a concern. In our 2015 study (Blatteau et al., 2015), the animals at risk weighed more than 350g: the mean weight was 380.5 g in the HW group and 309g in the LW group, a difference of more than 20% was noticed. In this study, all the animals can in fact be considered as being at risk since they all weigh more than 370 g, and animals with a slightly higher risk of DCS would be our controls. Actually, the risk factor linked to weight is generally associated with greater bullar birth in the presence of adipose tissue.

Although our results highlight various differences between Ctrl and non-DCS animals, we have not directly questioned the relationship between Ctrl and DCS animals, because hyperbaric exposure is a mandatory prerequisite to DCS. It is therefore probable that not all of these differences are directly related to DCS and that some may represent collateral modifications only.

We have mentioned “under” or “overexpression” suggesting a higher or a less absorption of metabolites, but it needs to be reminded that the compounds identified here are the result of the exogenous digestion (food bolus) and the endogenous digestion (including among other things debris from cells of the digestive system) substrates, which are themselves concerned with microbial activity, and that as such it remains difficult to identify their origin correctly (Chevalier et al., 2015). Characterizing the microbial community would partially clarify this point.

During any dive with compressed air the oxygen content in the whole-body increases. The amount of this increment depends on depth and dive duration. As the colon will be exposed to a higher content of oxygen, this can interact with the anaerobe gut bacteria and, indirectly, have some influence on the metabolomic fingerprint. Hyperoxia can indeed disrupt physiological reactions as presented before but, more generally, the oxygen molecule can also react chemically (due to its electronegativity) with a great number of molecules by attacking, for example, proteins with SH groups or the double bonds of unsaturated fatty acid chains. This process obviously applies to the molecules assayed in this work and it is possible to suspect a greater impact for oxygen in this protocol. However, this protocol is not intended to test the effects of hyperbaric oxygen per se: there is no normoxia hyperbaric control group.

From the point of view of clinical practice, it would be interesting to assess how much the reshuffle of the cecal metabolome affects the DCS occurence, and consequently whether a resetting of the Res metabolome into the Std one could be provocative. As a result, it could also impact DCS therapeutic with the aim to avoid degrading microbiota community, by limiting the side effects of hyperbaric oxygen therapy on anerobic species for example.

## Conclusion

The metabolomic analysis performed in diver rats shows a panel of 25 compounds whose expression differs from controls and which concerns biliary acids, primary and secondary biliary acids. There seems to be also weak differences in allocations of compound dedicated to various energy pathways, including (quasi) hormone. The availability of structural elements of cells and other micro-organisms is also different. These elements, probably resulting from the process of catabolism or anabolism of the host/microbial community couple, can participate in the growth or the formation of bacterial walls, for instance that finally suggest a different organization of the microbial community. Some of the constituent metabolites may also have a role in regulating inflammation, while others can be consumed for the benefit of oxidative stress management. Metabolomics offers a panoramic view that knows its limits. And although this conclusion should be considered with caution, a bundle of arguments seems to lead the interpretation of the results towards an oxidative stress contained by an appropriate inflammatory reaction, the whole being consecutive to the hyperbaric exposure. Each metabolite deserves special investigation.

## Data Availability

The original contributions presented in the study are included in the article/Supplementary Material, further inquiries can be directed to the corresponding author.

## Glossary

6-MP: 6-mercaptopurine
AHC: ascending hierarchical clustering
ALA: a-lipoic Acid
AMP: adenosine-monophosphate
ANOVA: analysis of variance
AR: altered ratio
CA: cholic acid
CDCA: chenodeoxycholic acid
CMP: cytidine monophosphate
Ctrl: control group
DCA: deoxycholic acid
DCS: decompression sickness
DHLCA: dehydrolithocholic acid
DHODH: dihydroorotate dehydrogenase
D*P*I: diet*pressure*pnteraction (in ANOVA)
FC: fold change
FRD: false rate discovery
GCAH: glycocholic acid hydrate
GMP: guanosine monophosphate
GPX: glutathione peroxidase
HSD: honestly significant difference
IL: interleukine
LCA: lithocholic acid
MAIZE: fed maize group
MCV: mean corpuscular volume
MIHP: motor impairment of the hind paw
MPS: motor performance score
MS: mass spectrography
Msw: meter of sea water
NAD: nicotinamide adénine dinucleotide
PAF: platelet activating factor
PCA: Principal component analysis
PLA: platelet
SFI index: sciatic functional impairment index
SOY: fed soy group
TBARS: Thiobarbituric Acid Reactive Susbtances
TRH: thyrotropin releasing hormone
TSH: thyrotropin stimuling hormone
UMP: uridine monophosphate
