# Supplementary material for: Cecal Metabolomic Fingerprint of Unscathed Rats: Does It Reflect the Good Response to a Provocative Decompression?
Source: Front Physiol. 2022 May 17;13:882944. doi: 10.3389/fphys.2022.882944 (PMC9152359; doi:10.3389/fphys.2022.882944)
Supplement: Supplementary file 1 [file DataSheet2.docx]

**Criteria of human end point**

| **degree of constraint from 0 (no constraint) to 3 (high stress)** | | | | |
| --- | --- | --- | --- | --- |
| Date : | Group : | Protocole : | | |
|  | Rat X | Rat Y | Rat Z | Rat… |
| **Behavior** |  | | | |
| Vocalize (shouting, crying) |  |  |  |  |
| withdrawn behavior |  |  |  |  |
| Agression behavior |  |  |  |  |
| Reduct° in exploratory behavior |  |  |  |  |
| Licking |  |  |  |  |
| Isolation from the group |  |  |  |  |
| **Eyes** |  | | | |
| Closed eyeslids |  |  |  |  |
| Sunken eyes |  |  |  |  |
| Tears |  |  |  |  |
| Bubles in the eyes |  |  |  |  |
| **Breath** |  | | | |
| High respiratory rate |  |  |  |  |
| Labored breathing |  |  |  |  |
| Gasps |  |  |  |  |
| Runny nose |  |  |  |  |
| **Appearance** |  | | | |
| Fur bristling |  |  |  |  |
| Arched back |  |  |  |  |
| Purple skin (hypoxya) |  |  |  |  |
| **Locomotion** |  | | | |
| Inhibited locomotion |  |  |  |  |
| Limping |  |  |  |  |
| Staggering gait |  |  |  |  |
| Lying down |  |  |  |  |
| Lying on its side |  |  |  |  |
| **Fore limbs** |  | | | |
| Atony |  |  |  |  |
| Paresis |  |  |  |  |
| Paralysis |  |  |  |  |
| **Right hind paw** |  | | | |
| Atony |  |  |  |  |
| Paresis |  |  |  |  |
| Paralysis |  |  |  |  |
| **Left hind paw** |  | | | |
| Atony |  |  |  |  |
| Paresis |  |  |  |  |
| Paralysis |  |  |  |  |
| **Convulsion** |  |  |  |  |
| **Pain during anesthesia** |  |  |  |  |
| **TOTAL SCORE:** |  |  |  |  |

According to our animal care committee, we used a system to determine when
the experiment must be stop, during the observation period. In all cases,
animals were continuously observed for 30 minutes after the end of the dive.

The table is inspired by the swiss veterinary guideline. The dedicated observer completes the form, given that a pain of degree 3 (very painful) in one case or a total amount of 12 are a stop point.

If sacrifice is needed, animal is first anesthetized with halothane (5% in
oxygen, Halothane, Belamont, France) and then sacrificed by injecting pentobarbital (200 mg/kg ip, Sanofi Santée, France). The gazeous anesthesia (halothane) is used in order to gain
time and to avoid to add another stress.
