# Supplementary material for: Cecal Metabolomic Fingerprint of Unscathed Rats: Does It Reflect the Good Response to a Provocative Decompression?
Source: Front Physiol. 2022 May 17;13:882944. doi: 10.3389/fphys.2022.882944 (PMC9152359; doi:10.3389/fphys.2022.882944)
Supplement: Supplementary file 2 [file DataSheet1.docx]

Supplementary data 1

| **Result** | **Metabolite** | **Pr > F** | **p-value Pressure** | **Fold-Change Diver/Control** | **p-value**  **Diet** | **Fold-Change MAIZE/SOY** |
| --- | --- | --- | --- | --- | --- | --- |
| D*P*I | 4-QUINOLINECARBOXYLIC ACID | 0.000 | 0.016915 | 0.84 | 0.00000 | 0.24 |
| D*P*I | 7-methylguanine | 0.000 | 0.025681 | 0.79 | 0.00000 | 0.11 |
| D*P*I | ARGININE | 0.000 | 0.004173 | 0.54 | 0.00038 | 0.45 |
| D*P*I | Gly-Gly | 0.000 | 0.019005 | 0.59 | 0.00000 | 0.28 |
| D*P*I | PYRROLE-2-CARBOXYLATE | 0.000 | 0.021722 | 0.56 | 0.00000 | 32.77 |
| D*P*I | THIOPURINE S-METHYLETHER | 0.000 | 0.022229 | 4.83 | 0.00817 | 0.13 |
| D*P*I | THYROTROPIN RELEASING HORMONE | 0.000 | 0.033936 | 0.57 | 0.00000 | 0.02 |
| P*I | C18_LysoPAF | 0.000 | 0.000001 | 2.09 | 0.76634 | 0.96 |
| P | ASPARTATE | 0.006 | 0.002067 | 1.39 | 0.64983 | 0.95 |
| P | CYTIDINE | 0.022 | 0.009361 | 0.66 | 0.13505 | 0.79 |
| P | glycocholic acid hydrate | 0.003 | 0.000335 | 1.97 | 0.50852 | 0.89 |
| P | L-Asparagine | 0.017 | 0.005391 | 1.41 | 0.50206 | 0.92 |
| P | RHAMNOSE | 0.040 | 0.023204 | 1.54 | 0.10623 | 0.74 |
| I | 5b-cholanic acid-3-ONE | 0.017 | 0.062888 | 0.84 | 0.47721 | 0.93 |
| I | DEOXYGUANOSINE-MONOPHOSPHATE | 0.019 | 0.919039 | 0.98 | 0.61697 | 1.10 |
| I | GLUCOSE 6-PHOSPHATE | 0.002 | 0.165031 | 0.73 | 0.11308 | 0.69 |
| I | GLYCEROL 3-PHOSPHATE | 0.005 | 0.261198 | 0.72 | 0.28514 | 0.73 |
| D*P | (\xB1)-a-Lipoic acid | 0.001 | 0.034508 | 4.61 | 0.01076 | 0.12 |
| D*P | ADENOSINE-MONOPHOSPHATE | 0.000 | 0.000776 | 1.90 | 0.00000 | 3.18 |
| D*P | CARNOSINE | 0.000 | 0.001480 | 0.49 | 0.00012 | 0.41 |
| D*P | CHENODEOXYCHOLATE | 0.000 | 0.037227 | 0.71 | 0.00016 | 1.97 |
| D*P | L-Glutamine | 0.000 | 0.033810 | 0.59 | 0.00002 | 0.29 |
| D*P | L-Leucine | 0.000 | 0.028156 | 0.74 | 0.00001 | 0.51 |
| D*P | METHYLTHIOADENOSINE | 0.000 | 0.023341 | 0.74 | 0.00000 | 0.32 |
| D*P | N-ACETYLGALACTOSAMINE | 0.000 | 0.001363 | 1.80 | 0.00069 | 1.87 |
| D*P | N-ACETYLNEURAMINATE | 0.000 | 0.000597 | 2.05 | 0.00000 | 3.68 |
| D*P | N-Acetylneuraminic acid | 0.000 | 0.047934 | 1.59 | 0.00016 | 2.70 |
| D*P | PROTOPORPHYRIN | 0.000 | 0.015469 | 0.75 | 0.00000 | 1.93 |
| D*P | PTERIN | 0.000 | 0.029561 | 0.64 | 0.00010 | 0.42 |
| D | (+)-Catechin hydrate | 0.011 | 0.181077 | 1.28 | 0.02030 | 0.65 |
| D | (2-AMINOETHYL)PHOSPHONATE | 0.003 | 0.296803 | 0.77 | 0.00028 | 0.36 |
| D | 14-0_LysoPE | 0.000 | 0.732475 | 1.09 | 0.00000 | 0.23 |
| D | 16:0_LysoPA | 0.003 | 0.099656 | 0.62 | 0.00091 | 0.34 |
| D | 16-0_LysoPE | 0.000 | 0.993670 | 1.00 | 0.00000 | 0.26 |
| D | 16-0_LysoPG | 0.000 | 0.147703 | 0.76 | 0.00000 | 0.35 |
| D | 16-0_LysoPI | 0.006 | 0.427934 | 0.75 | 0.00062 | 0.21 |
| D | 17a-Ethynylestradiol | 0.000 | 0.118138 | 0.87 | 0.00000 | 2.51 |
| D | 17a-Hydroxyprogesterone | 0.005 | 0.184236 | 0.85 | 0.00079 | 0.64 |
| D | 18-1_LysoPA | 0.045 | 0.742986 | 0.90 | 0.00536 | 0.35 |
| D | 18-1_LysoPE | 0.000 | 0.455495 | 1.13 | 0.00003 | 0.45 |
| D | 1-METHYLADENOSINE | 0.005 | 0.864207 | 1.03 | 0.00096 | 0.58 |
| D | 1-METHYL-L-HISTIDINE | 0.025 | 0.051539 | 1.34 | 0.01878 | 1.43 |
| D | 1-Methyluric acid | 0.009 | 0.971751 | 1.01 | 0.00081 | 0.23 |
| D | 2,6-quinolinediol | 0.000 | 0.647492 | 0.85 | 0.00000 | 178.32 |
| D | 3-AMINO-4-HYDROXYBENZOATE | 0.000 | 0.588934 | 0.90 | 0.00000 | 12.38 |
| D | 3-HYDROXYANTHRANILATE | 0.000 | 0.506459 | 0.88 | 0.00000 | 12.84 |
| D | 4-IMIDAZOLEACETATE | 0.007 | 0.088896 | 2.31 | 0.00802 | 4.39 |
| D | 5b-cholanic acid-3a-OL-12-ONE | 0.000 | 0.487034 | 0.82 | 0.00007 | 3.89 |
| D | 5b-cholanic acid-3a-OL-7,12-DIONE | 0.000 | 0.789038 | 0.98 | 0.00000 | 1.72 |
| D | 5'-DEOXYADENOSINE | 0.000 | 0.890395 | 0.96 | 0.00002 | 0.13 |
| D | 5-hydroxyindole-3-acetic acid | 0.000 | 0.503034 | 1.16 | 0.00000 | 6.64 |
| D | ADENOSINE | 0.000 | 0.230700 | 0.80 | 0.00002 | 0.40 |
| D | BETA-ALANINE | 0.001 | 0.078335 | 0.62 | 0.00030 | 0.33 |
| D | BETA-NICOTINAMIDE ADENINE DINUCLEOTIDE | 0.009 | 0.824158 | 1.06 | 0.00309 | 0.42 |
| D | CELLOBIOSE | 0.018 | 0.106161 | 0.70 | 0.00594 | 0.53 |
| D | CHOLATE | 0.000 | 0.500183 | 0.93 | 0.00000 | 3.37 |
| D | CORTEXOLONE | 0.000 | 0.308736 | 1.43 | 0.00000 | 52.53 |
| D | CYSTATHIONINE | 0.030 | 0.227727 | 1.76 | 0.03581 | 0.35 |
| D | CYSTEATE | 0.000 | 0.117401 | 1.44 | 0.00000 | 0.25 |
| D | CYSTEINE | 0.019 | 0.051060 | 0.53 | 0.02349 | 2.14 |
| D | CYTIDINE MONOPHOSPHATE | 0.030 | 0.813084 | 1.06 | 0.00362 | 0.46 |
| D | DEOXYADENOSINE MONOPHOSPHATE | 0.015 | 0.802912 | 0.93 | 0.00238 | 0.37 |
| D | DEOXYGUANOSINE | 0.001 | 0.608261 | 1.14 | 0.00012 | 3.21 |
| D | DIPALMITOYLGLYCEROL | 0.000 | 0.076276 | 0.71 | 0.00000 | 0.00 |
| D | D-ORNITHINE | 0.002 | 0.415139 | 1.17 | 0.00203 | 0.53 |
| D | GALACTITOL | 0.000 | 0.143402 | 0.86 | 0.00007 | 0.63 |
| D | GALACTOSAMINE | 0.000 | 0.305340 | 0.81 | 0.00001 | 0.34 |
| D | GALACTOSE | 0.002 | 0.687560 | 0.92 | 0.00018 | 0.41 |
| D | GLYCINE | 0.000 | 0.678738 | 0.95 | 0.00002 | 0.52 |
| D | GUANINE | 0.037 | 0.812269 | 1.05 | 0.00574 | 1.76 |
| D | HIPPURATE | 0.000 | 0.267146 | 0.69 | 0.00000 | 157.15 |
| D | HISTIDINE | 0.008 | 0.063139 | 0.82 | 0.00551 | 0.74 |
| D | HYPOTAURINE | 0.006 | 0.082473 | 2.30 | 0.00824 | 4.14 |
| D | HYPOXANTHINE | 0.004 | 0.102436 | 0.73 | 0.00093 | 0.50 |
| D | INDOLE-3-ACETATE | 0.000 | 0.531423 | 1.12 | 0.00000 | 32.36 |
| D | INOSINE | 0.000 | 0.394235 | 0.86 | 0.00000 | 0.30 |
| D | LACTOSE | 0.012 | 0.181366 | 0.54 | 0.00220 | 0.17 |
| D | LITHOCHOLATE | 0.000 | 0.859140 | 0.95 | 0.00007 | 3.62 |
| D | L-Threo-3-Phenylserine (DL-3-Phenylserine) | 0.000 | 0.949335 | 1.01 | 0.00000 | 4.12 |
| D | L-tyrosine | 0.002 | 0.337470 | 0.89 | 0.00063 | 0.64 |
| D | MALTOSE | 0.029 | 0.191824 | 0.69 | 0.00649 | 0.44 |
| D | MANNITOL | 0.000 | 0.072932 | 1.58 | 0.00004 | 0.29 |
| D | N6-(DELTA2-ISOPENTENYL)-ADENINE | 0.000 | 0.873562 | 0.99 | 0.00000 | 0.39 |
| D | N-ACETYLASPARTATE | 0.021 | 0.364345 | 0.85 | 0.00308 | 1.74 |
| D | NERVONATE | 0.000 | 0.538488 | 1.10 | 0.00000 | 0.26 |
| D | PHENYLALANINE | 0.000 | 0.295369 | 0.83 | 0.00004 | 0.43 |
| D | PHOSPHORYLCHOLINE | 0.017 | 0.858690 | 1.09 | 0.00245 | 0.12 |
| D | Pro-Leu | 0.006 | 0.198900 | 0.80 | 0.00085 | 0.54 |
| D | RIBITOL | 0.010 | 0.244591 | 1.64 | 0.00756 | 0.27 |
| D | RIBOFLAVIN | 0.019 | 0.132877 | 1.30 | 0.00616 | 0.60 |
| D | RIBOSE | 0.000 | 0.552413 | 0.93 | 0.00000 | 0.29 |
| D | S-ADENOSYLMETHIONINE | 0.000 | 0.086765 | 0.72 | 0.00000 | 0.20 |
| D | S-HEXYL-GLUTATHIONE | 0.000 | 0.978366 | 1.00 | 0.00000 | 0.21 |
| D | SHIKIMATE | 0.000 | 0.360045 | 0.84 | 0.00001 | 0.37 |
| D | STACHYOSE | 0.026 | 0.519998 | 0.87 | 0.00382 | 1.95 |
| D | THIAMINE MONOPHOSPHATE | 0.012 | 0.441104 | 1.23 | 0.00188 | 0.39 |
| D | THYMIDINE-MONOPHOSPHATE | 0.003 | 0.189384 | 0.70 | 0.00096 | 0.37 |
| D | trans-3-Hydroxycinnamic acid (m-Coumaric acid) | 0.000 | 0.391325 | 0.87 | 0.00000 | 0.00 |
| D | TRICOSANOATE | 0.001 | 0.079588 | 1.44 | 0.00352 | 0.53 |
| D | TRYPTOPHAN | 0.000 | 0.514199 | 0.92 | 0.00000 | 0.46 |
| D | URACIL 5-CARBOXYLATE | 0.009 | 0.158574 | 0.72 | 0.00203 | 2.20 |
| D | UREIDOPROPIONATE | 0.002 | 0.579935 | 0.87 | 0.00021 | 0.33 |
| D | URIDINE | 0.000 | 0.413112 | 0.84 | 0.00001 | 0.31 |
| D | URIDINE 5'-DIPHOSPHATE | 0.039 | 0.300569 | 0.58 | 0.00825 | 5.59 |
| D | URIDINE DIPHOSPHATE GLUCOSE | 0.040 | 0.242693 | 0.54 | 0.01055 | 4.95 |
| D | VITAMIN D2 | 0.000 | 0.801378 | 1.08 | 0.00000 | 0.15 |
| D | XANTHOSINE | 0.001 | 0.571578 | 1.20 | 0.00009 | 0.19 |
| D | XANTHURENATE | 0.000 | 0.264313 | 0.91 | 0.00000 | 0.12 |
| D*I | 25-HYDROXYCHOLESTEROL | 0.000 | 0.806121 | 0.97 | 0.00027 | 1.65 |
| D*I | 4-GUANIDINOBUTANOATE | 0.000 | 0.172833 | 0.74 | 0.00007 | 0.37 |
| D*I | D-RIBOSE 5-PHOSPHATE | 0.000 | 0.161263 | 0.73 | 0.00544 | 0.52 |
| D*I | PYRIDOXAL | 0.000 | 0.957266 | 1.01 | 0.00061 | 1.84 |
| NS | 14-0_LysoPC | 0.504 | 0.367100 | 0.72 | 0.25843 | 0.66 |
| NS | 16-0_LysoPC | 0.448 | 0.174065 | 0.76 | 0.50110 | 1.14 |
| NS | 16-0_LysoPS | 0.113 | 0.107407 | 3.60 | 0.29663 | 2.14 |
| NS | 18-0_LysoPC | 0.540 | 0.824382 | 0.95 | 0.16187 | 0.73 |
| NS | 18-0_LysoPE | 0.052 | 0.487502 | 1.21 | 0.01409 | 0.49 |
| NS | 18-1_LysoPC | 0.226 | 0.068226 | 0.69 | 0.29994 | 0.81 |
| NS | 18-1_LysoPS | 0.319 | 0.904019 | 1.06 | 0.17989 | 2.07 |
| NS | 2-DEOXY-D-GLUCOSE | 0.204 | 0.256592 | 3.15 | 0.17421 | 0.23 |
| NS | 2-hydroxycaproic acid | 0.157 | 0.029171 | 6.74 | 0.93859 | 1.05 |
| NS | 3-b-Hydroxyandrost-5-en-17-one (Dehydroepiandrosterone) | 0.072 | 0.181534 | 0.90 | 0.03344 | 0.84 |
| NS | 4 carbamyl-1-methylpyridinium | 0.297 | 0.882917 | 1.02 | 0.05988 | 0.73 |
| NS | 5b-cholanic acid-3a,12a-DIOL-7-ONE | 0.215 | 0.874090 | 0.89 | 0.04022 | 7.51 |
| NS | 5-HYDROXYMETHYLURACIL | 0.078 | 0.528431 | 1.22 | 0.07187 | 0.56 |
| NS | 5-Hydroxynicotinic acid | 0.067 | 0.493033 | 0.92 | 0.03805 | 0.77 |
| NS | AGMATINE SULFATE | 0.352 | 0.614662 | 0.87 | 0.47344 | 0.82 |
| NS | ALLANTOIN | 0.219 | 0.463348 | 1.19 | 0.17742 | 1.39 |
| NS | ALPHA-D-GLUCOSE | 0.193 | 0.684230 | 0.94 | 0.11210 | 0.77 |
| NS | ANSERINE | 0.071 | 0.012145 | 0.21 | 0.39680 | 0.65 |
| NS | ARABINOSE | 0.739 | 0.344180 | 1.22 | 0.83756 | 0.96 |
| NS | ASPARAGINE | 0.773 | 0.994029 | 1.00 | 0.30038 | 0.78 |
| NS | BILIVERDIN | 0.146 | 0.550638 | 1.20 | 0.02818 | 0.49 |
| NS | BIOTIN | 0.108 | 0.045432 | 1.44 | 0.16752 | 0.78 |
| NS | C16 | 0.583 | 0.450731 | 0.70 | 0.56905 | 0.77 |
| NS | C16_PlasmLPC | 0.567 | 0.393688 | 0.68 | 0.67347 | 0.83 |
| NS | C18 | 0.193 | 0.293708 | 0.65 | 0.06398 | 0.46 |
| NS | CADAVERINE | 0.647 | 0.231606 | 1.30 | 0.67235 | 0.91 |
| NS | CITRULLINE | 0.121 | 0.162786 | 0.90 | 0.60369 | 1.04 |
| NS | CORTISOL | 0.042 | 0.060218 | 0.56 | 0.10431 | 1.65 |
| NS | CORTISOL 21-ACETATE | 0.324 | 0.095161 | 1.27 | 0.58488 | 0.93 |
| NS | CREATINE | 0.352 | 0.468751 | 0.69 | 0.73560 | 0.85 |
| NS | DEOXYADENOSINE | 0.261 | 0.926608 | 1.03 | 0.04892 | 1.96 |
| NS | DEOXYCHOLATE | 0.174 | 0.335756 | 0.86 | 0.05272 | 1.38 |
| NS | DEOXYCYTIDINE | 0.839 | 0.884440 | 0.98 | 0.65850 | 0.94 |
| NS | DEOXYCYTIDINE MONOPHOSPHATE | 0.310 | 0.918751 | 0.97 | 0.06111 | 0.58 |
| NS | DPPC | 0.192 | 0.065232 | 0.62 | 0.46067 | 1.21 |
| NS | D-Raffinose (D-(+)-Raffinose pentahydrate ) | 0.142 | 0.030151 | 0.63 | 0.54995 | 0.88 |
| NS | GLUCOSAMINE 6-SULFATE | 0.102 | 0.027613 | 2.27 | 0.93946 | 1.03 |
| NS | GLUCURONATE | 0.183 | 0.037205 | 1.45 | 0.55331 | 0.90 |
| NS | GLUTAMATE | 0.440 | 0.723812 | 0.96 | 0.11302 | 1.20 |
| NS | GLYCEROL-MYRISTATE | 0.497 | 0.392090 | 0.79 | 0.56606 | 0.85 |
| NS | Gly-Pro | 0.272 | 0.078575 | 0.71 | 0.36087 | 0.84 |
| NS | GUANOSINE | 0.277 | 0.601062 | 0.93 | 0.06744 | 0.76 |
| NS | HOMOSERINE | 0.635 | 0.588505 | 1.15 | 0.24286 | 0.74 |
| NS | KYNURENINE | 0.059 | 0.021684 | 0.45 | 0.12797 | 0.60 |
| NS | L-carnitine hydrochloride | 0.188 | 0.078001 | 0.73 | 0.32135 | 0.84 |
| NS | L-Methionine | 0.315 | 0.941626 | 0.99 | 0.06708 | 1.21 |
| NS | L-Serine | 0.195 | 0.566287 | 1.07 | 0.04209 | 0.78 |
| NS | LYSINE | 0.788 | 0.909300 | 1.02 | 0.54274 | 0.92 |
| NS | LYXOSE | 0.500 | 0.387658 | 1.20 | 0.42496 | 1.19 |
| NS | MELATONIN | 0.158 | 0.849605 | 1.05 | 0.11153 | 0.68 |
| NS | MYOINOSITOL | 0.895 | 0.832251 | 1.03 | 0.64255 | 1.08 |
| NS | N-ACETYLASPARAGINE | 0.405 | 0.577437 | 1.21 | 0.64242 | 0.85 |
| NS | N-Acetyl-D-sphingosine | 0.704 | 0.617723 | 0.88 | 0.32592 | 0.77 |
| NS | N-ACETYLGLUCOSAMINE | 0.064 | 0.378799 | 1.11 | 0.02928 | 1.29 |
| NS | N-ALPHA-ACETYLLYSINE | 0.337 | 0.414849 | 1.16 | 0.10020 | 1.36 |
| NS | OXOGLUTARATE | 0.579 | 0.284922 | 1.35 | 0.50056 | 1.21 |
| NS | PANTOTHENATE | 0.797 | 0.435127 | 1.20 | 0.54011 | 0.87 |
| NS | PHOSPHOENOLPYRUVATE | 0.108 | 0.393366 | 1.79 | 0.25467 | 0.45 |
| NS | PIPECOLATE | 0.503 | 0.799048 | 0.94 | 0.27797 | 0.78 |
| NS | pregnenolone sulfate | 0.374 | 0.330324 | 1.44 | 0.21441 | 1.61 |
| NS | PUTRESCINE | 0.085 | 0.827288 | 1.04 | 0.05212 | 0.67 |
| NS | PYROGLUTAMATE | 0.662 | 0.233418 | 0.81 | 0.76704 | 1.05 |
| NS | QUINATE | 0.132 | 0.874850 | 1.03 | 0.02098 | 0.61 |
| NS | SACCHARATE | 0.067 | 0.271705 | 1.29 | 0.01971 | 0.57 |
| NS | SORBOSE | 0.074 | 0.890665 | 1.02 | 0.01010 | 0.63 |
| NS | SPHINGANINE | 0.127 | 0.754922 | 0.96 | 0.02898 | 1.38 |
| NS | taurocholic acid (iso1) | 0.151 | 0.039165 | 4.43 | 0.67149 | 1.29 |
| NS | THYMIDINE | 0.107 | 0.832415 | 0.97 | 0.01472 | 0.66 |
| NS | THYMINE | 0.066 | 0.162483 | 1.47 | 0.09259 | 1.59 |
| NS | TRYPTAMINE | 0.268 | 0.618877 | 1.18 | 0.07621 | 0.54 |
| NS | URIDINE DIPHOSPHATE-N-ACETYLGLUCOSAMINE | 0.248 | 0.512963 | 0.81 | 0.64655 | 1.16 |
| NS | URIDINE MONOPHOSPHATE | 0.645 | 0.498612 | 0.81 | 0.79667 | 0.92 |
| NS | XANTHINE | 0.373 | 0.247487 | 0.81 | 0.21873 | 0.80 |

Supplementary data 2

| **Comparison of metabolite lists** | |
| --- | --- |
| 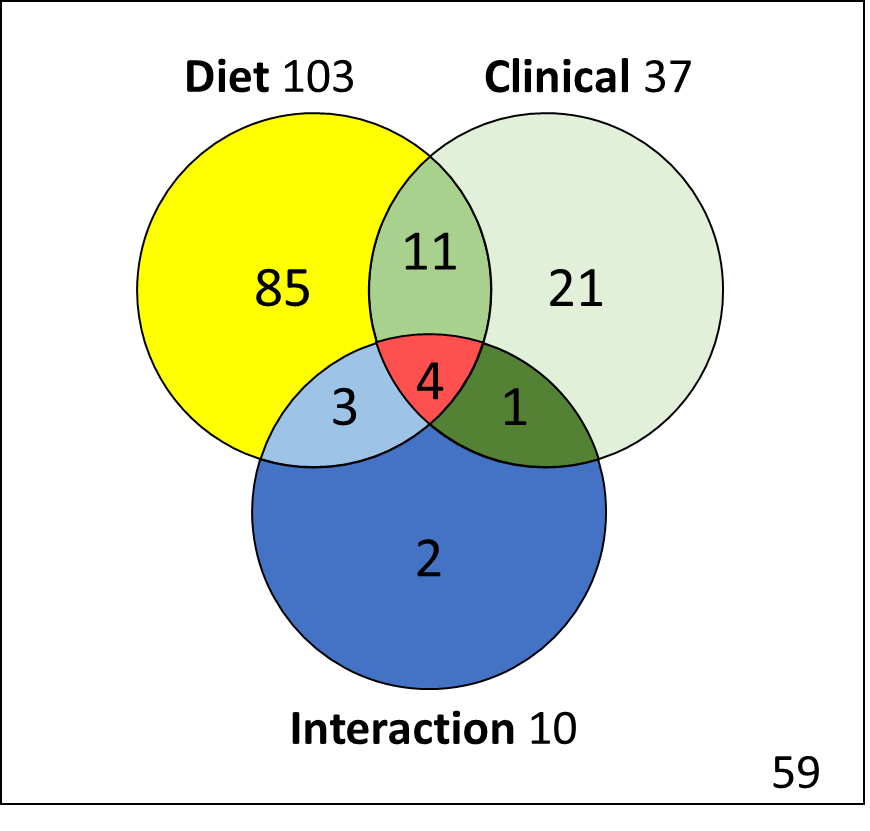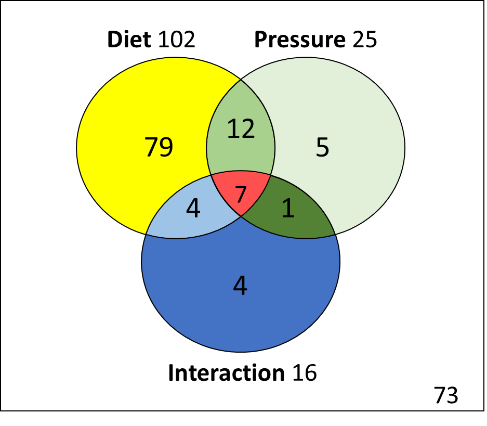 **Venn diagram of the present study** | **Venn diagram of the previous study (deMaistre et al, 2020)** |
|  |  |
| n=112 | n=127 |
| (\xB1)-a-Lipoic acid | (\xB1)-a-Lipoic acid |
| (+)-Catechin hydrate | (+)-Catechin hydrate |
| (2-AMINOETHYL)PHOSPHONATE | (2-AMINOETHYL)PHOSPHONATE |
|  | 14-0_LysoPC |
| 14-0_LysoPE | 14-0_LysoPE |
| 16:0_LysoPA |  |
|  | 16-0_LysoPC |
| 16-0_LysoPE | 16-0_LysoPE |
| 16-0_LysoPG | 16-0_LysoPG |
| 16-0_LysoPI | 16-0_LysoPI |
| 17a-Ethynylestradiol | 17a-Ethynylestradiol |
| 17a-Hydroxyprogesterone | 17a-Hydroxyprogesterone |
|  | 18-0_LysoPC |
|  | 18-0_LysoPE |
| 18-1_LysoPA | 18-1_LysoPA |
|  | 18-1_LysoPC |
| 18-1_LysoPE | 18-1_LysoPE |
| 1-METHYLADENOSINE | 1-METHYLADENOSINE |
| 1-METHYL-L-HISTIDINE | 1-METHYL-L-HISTIDINE |
| 1-Methyluric acid | 1-Methyluric acid |
| 2,6-quinolinediol | 2,6-quinolinediol |
| 25-HYDROXYCHOLESTEROL | 25-HYDROXYCHOLESTEROL |
|  | 2-hydroxycaproic acid |
| 3-AMINO-4-HYDROXYBENZOATE | 3-AMINO-4-HYDROXYBENZOATE |
| 3-HYDROXYANTHRANILATE | 3-HYDROXYANTHRANILATE |
| 4-GUANIDINOBUTANOATE | 4-GUANIDINOBUTANOATE |
| 4-IMIDAZOLEACETATE | 4-IMIDAZOLEACETATE |
| 4-QUINOLINECARBOXYLIC ACID | 4-QUINOLINECARBOXYLIC ACID |
|  | 5b-cholanic acid-3a,12a-DIOL-7-ONE |
| 5b-cholanic acid-3a-OL-12-ONE | 5b-cholanic acid-3a-OL-12-ONE |
| 5b-cholanic acid-3a-OL-7,12-DIONE | 5b-cholanic acid-3a-OL-7,12-DIONE |
| 5b-cholanic acid-3-ONE |  |
| 5'-DEOXYADENOSINE | 5'-DEOXYADENOSINE |
|  | 5-hydroxyindole-3-acetic acid |
|  | 5-HYDROXYMETHYLURACIL |
| 5-hydroxyindole-3-acetic acid | 5-Hydroxynicotinic acid |
| 7-methylguanine | 7-methylguanine |
| ADENOSINE | ADENOSINE |
| ADENOSINE-MONOPHOSPHATE | ADENOSINE-MONOPHOSPHATE |
|  | ANSERINE |
| ARGININE |  |
| ASPARTATE |  |
| BETA-ALANINE | BETA-ALANINE |
| BETA-NICOTINAMIDE ADENINE DINUCLEOTIDE | BETA-NICOTINAMIDE ADENINE DINUCLEOTIDE |
|  | C16 |
|  | C16_PlasmLPC |
|  | C18 |
| C18_LysoPAF | C18_LysoPAF |
| CARNOSINE | CARNOSINE |
| CELLOBIOSE |  |
| CHENODEOXYCHOLATE |  |
| CHOLATE | CHOLATE |
|  | CITRULLINE |
| CORTEXOLONE | CORTEXOLONE |
|  | CREATINE |
| CYSTATHIONINE | CYSTATHIONINE |
| CYSTEATE | CYSTEATE |
| CYSTEINE | CYSTEINE |
| CYTIDINE |  |
| CYTIDINE MONOPHOSPHATE | CYTIDINE MONOPHOSPHATE |
| DEOXYADENOSINE MONOPHOSPHATE | DEOXYADENOSINE MONOPHOSPHATE |
|  | DEOXYCYTIDINE MONOPHOSPHATE |
| DEOXYGUANOSINE | DEOXYGUANOSINE |
| DEOXYGUANOSINE-MONOPHOSPHATE |  |
| DIPALMITOYLGLYCEROL | Dipalmitoylglycerol |
| D-ORNITHINE | D-ORNITHINE |
|  | DPPC |
| D-RIBOSE 5-PHOSPHATE | D-RIBOSE 5-PHOSPHATE |
| GALACTITOL | GALACTITOL |
| GALACTOSAMINE | GALACTOSAMINE |
| GALACTOSE |  |
| GLUCOSE 6-PHOSPHATE | GLUCOSE 6-PHOSPHATE |
| GLYCEROL 3-PHOSPHATE |  |
|  | GLYCEROL-MYRISTATE |
| GLYCINE | GLYCINE |
| glycocholic acid hydrate |  |
| Gly-Gly | Gly-Gly |
|  | Gly-Pro |
| GUANINE | GUANINE |
|  | GUANOSINE |
| HIPPURATE | Hippurate |
| HISTIDINE | HISTIDINE |
| HYPOTAURINE | HYPOTAURINE |
| HYPOXANTHINE | HYPOXANTHINE |
| INDOLE-3-ACETATE | INDOLE-3-ACETATE |
| INOSINE | INOSINE |
|  | KYNURENINE |
| LACTOSE |  |
| L-Asparagine |  |
|  | L-carnitine hydrochloride |
| L-Glutamine | L-Glutamine |
| LITHOCHOLATE | LITHOCHOLATE |
| L-Leucine | L-Leucine |
|  | L-Methionine |
|  | L-Serine |
| L-Threo-3-Phenylserine (DL-3-Phenylserine) | L-Threo-3-Phenylserine (DL-3-Phenylserine) |
| L-tyrosine | L-tyrosine |
| MALTOSE |  |
| MANNITOL | MANNITOL |
| METHYLTHIOADENOSINE | METHYLTHIOADENOSINE |
|  | MYOINOSITOL |
| N6-(DELTA2-ISOPENTENYL)-ADENINE | N6-(DELTA2-ISOPENTENYL)-ADENINE |
|  | N-ACETYLASPARAGINE |
| N-ACETYLASPARTATE | N-ACETYLASPARTATE |
| N-ACETYLGALACTOSAMINE | N-ACETYLGALACTOSAMINE |
|  | N-ACETYLGLUCOSAMINE |
| N-ACETYLNEURAMINATE | N-ACETYLNEURAMINATE |
| N-Acetylneuraminic acid | N-Acetylneuraminic acid |
| NERVONATE | NERVONATE |
| PHENYLALANINE | PHENYLALANINE |
|  | PHOSPHOENOLPYRUVATE |
| PHOSPHORYLCHOLINE | PHOSPHORYLCHOLINE |
|  | PIPECOLATE |
| Pro-Leu |  |
| PROTOPORPHYRIN | PROTOPORPHYRIN |
| PTERIN | PTERIN |
|  | PUTRESCINE |
| PYRIDOXAL | PYRIDOXAL |
|  | PYROGLUTAMATE |
| PYRROLE-2-CARBOXYLATE | PYRROLE-2-CARBOXYLATE |
| RHAMNOSE |  |
| RIBITOL | RIBITOL |
| RIBOFLAVIN | RIBOFLAVIN |
| RIBOSE | RIBOSE |
| S-ADENOSYLMETHIONINE | S-ADENOSYLMETHIONINE |
| S-HEXYL-GLUTATHIONE | S-HEXYL-GLUTATHIONE |
| SHIKIMATE | SHIKIMATE |
| STACHYOSE |  |
| THIAMINE MONOPHOSPHATE | THIAMINE MONOPHOSPHATE |
| THIOPURINE S-METHYLETHER | THIOPURINE S-METHYLETHER |
|  | THYMIDINE |
| THYMIDINE-MONOPHOSPHATE | THYMIDINE-MONOPHOSPHATE |
| THYROTROPIN RELEASING HORMONE | THYROTROPIN RELEASING HORMONE |
| trans-3-Hydroxycinnamic acid (m-Coumaric acid) | trans-3-Hydroxycinnamic acid (m-Coumaric acid) |
| TRICOSANOATE | TRICOSANOATE |
| TRYPTOPHAN | TRYPTOPHAN |
| URACIL 5-CARBOXYLATE | URACIL 5-CARBOXYLATE |
| UREIDOPROPIONATE | UREIDOPROPIONATE |
| URIDINE |  |
| URIDINE 5'-DIPHOSPHATE | URIDINE 5'-DIPHOSPHATE |
| URIDINE DIPHOSPHATE GLUCOSE | URIDINE DIPHOSPHATE GLUCOSE |
| VITAMIN D2 | VITAMIN D2 |
|  | XANTHINE |
| XANTHOSINE | XANTHOSINE |
| XANTHURENATE | XANTHURENATE |
